# Supplementary material for: Exceptional uranium(VI)-nitride triple bond covalency from 15N nuclear magnetic resonance spectroscopy and quantum chemical analysis
Source: Nat Commun. 2021 Sep 24;12:5649. doi: 10.1038/s41467-021-25863-2 (PMC8463702; doi:10.1038/s41467-021-25863-2)
Supplement: Supplementary file 1 — Supplementary Information [file 41467_2021_25863_MOESM1_ESM.pdf]

Exceptional Uranium(VI)-Nitride Triple Bond Covalency from ^{15}N Nuclear Magnetic Resonance Spectroscopy and Quantum Chemical Analysis

Jingzhen Du,[a] John A. Seed,[a] Victoria E. J. Berryman,[a] Nikolas Kaltsoyannis,[a]

Ralph W. Adams,*[a] Daniel Lee,*[b] and Stephen T. Liddle*[a]

[a] Department of Chemistry, The University of Manchester, Oxford Road, Manchester, M13 9PL, UK. [b] Department of Chemical Engineering and Analytical Science, The University of Manchester, Manchester, M13 9PL, UK.

*For correspondence: ralph.adams@manchester.ac.uk; daniel.lee@manchester.ac.uk; steve.liddle@manchester.ac.uk

The following codes are used in the δ_{iso} vs bond order graphs:

**Code**	**Formula**
**1***	[U(N*)(Tren^{TIPS})]
**VN(O)**	[V(N)(L^{MeDipp})(ODipp))]
**VN(N)**	[V(N)(L^{MeDipp}){N(Tol)(Mes)}]
**TiN**	[Ti(N)(NP)$_2$]$^{1-}$
**TiNK**	[Ti(μ-N)(NP)$_2$K(18C6)]
**MoN**	[Mo(N){N(Bu^t)(C$_6$H$_3$-3,5-Me$_2$)}$_3$]
**MoNSnCl$_2$**	[Mo(NSnCl$_2$){N(Bu^t)(C$_6$H$_3$-3,5-Me$_2$)}$_3$]
**MoNGeCl$_2$**	[Mo(NGeCl$_2$){N(Bu^t)(C$_6$H$_3$-3,5-Me$_2$)}$_3$]
**MoNBF$_3$**	[Mo(NBF$_3$){N(Bu^t)(C$_6$H$_3$-3,5-Me$_2$)}$_3$]
**MoNSiMe$_3$**	[Mo(NSiMe$_3$){N(Bu^t)(C$_6$H$_3$-3,5-Me$_2$)}$_3$]
**MoNCOPh**	[Mo(NCOPh){N(Bu^t)(C$_6$H$_3$-3,5-Me$_2$)}$_3$]
**MoNEt**	[Mo(NEt){N(Bu^t)(C$_6$H$_3$-3,5-Me$_2$)}$_3$]
**MoNCH$_2$**	[Mo(NCH$_2$){N(Bu^t)(C$_6$H$_3$-3,5-Me$_2$)}$_3$]
**TiNH**	[Ti(NH)(L^{ButDipp})(NTol$_2$)]
**ZrNH**	[Zr(NH)(NP)$_2$]
**ThNTh**	[(N")$_3$Th(μ-N)Th(N")$_3$][K(18C6)(THF)$_2$]
**ThNH$_2$**	[Th(NH$_2$)(N")$_3$]

Supplementary Figures

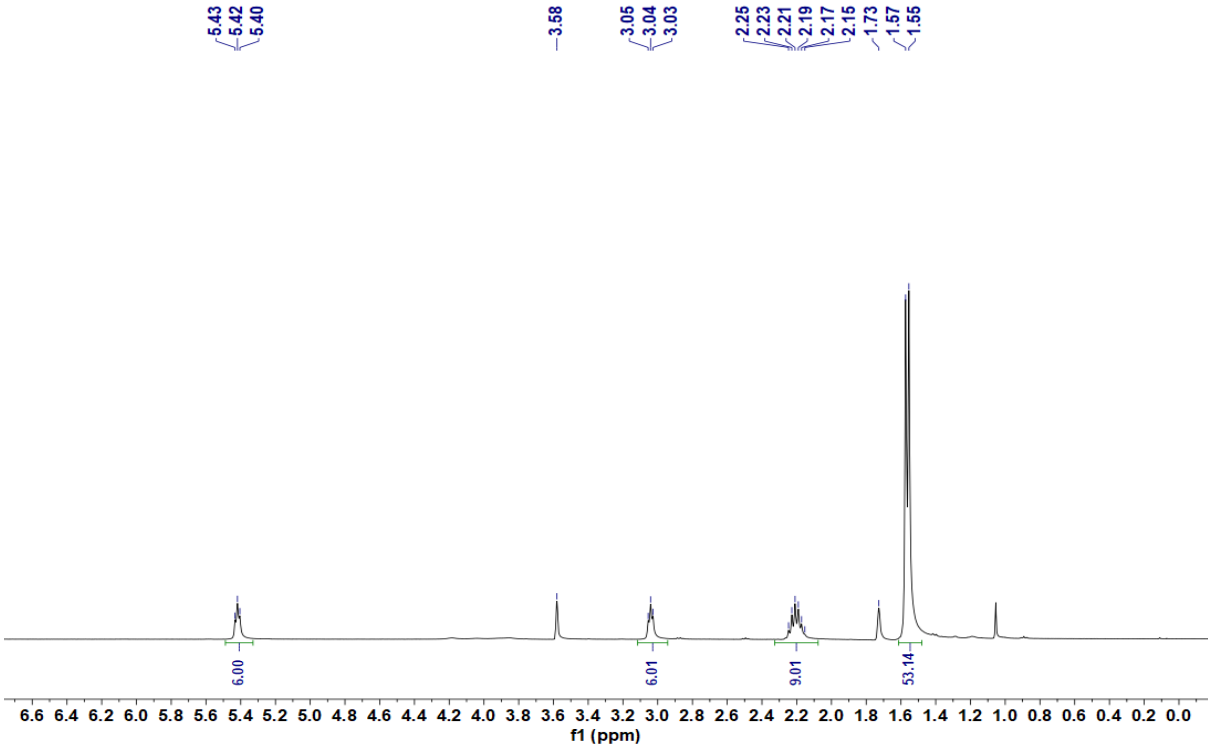

Supplementary Figure 1. ^{1}H NMR (400 MHz, D_8-THF, 298 K) of **1***.

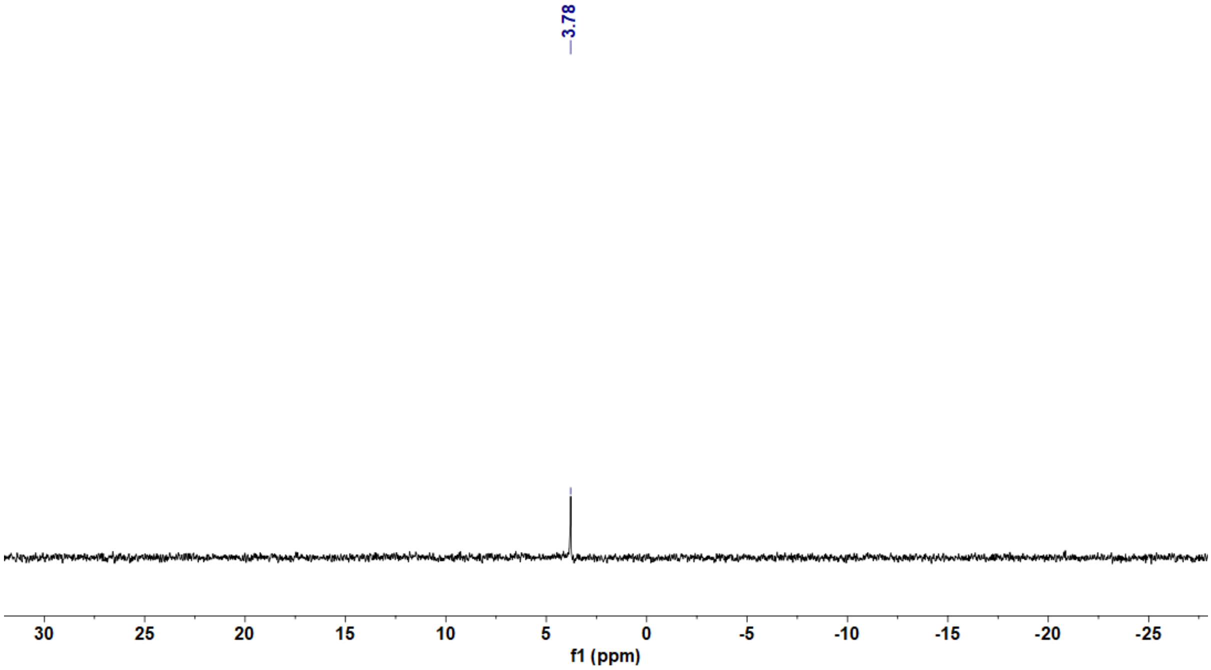

Supplementary Figure 2. ^{29}Si NMR (79 MHz, D_8-THF, 298 K) of **1***.

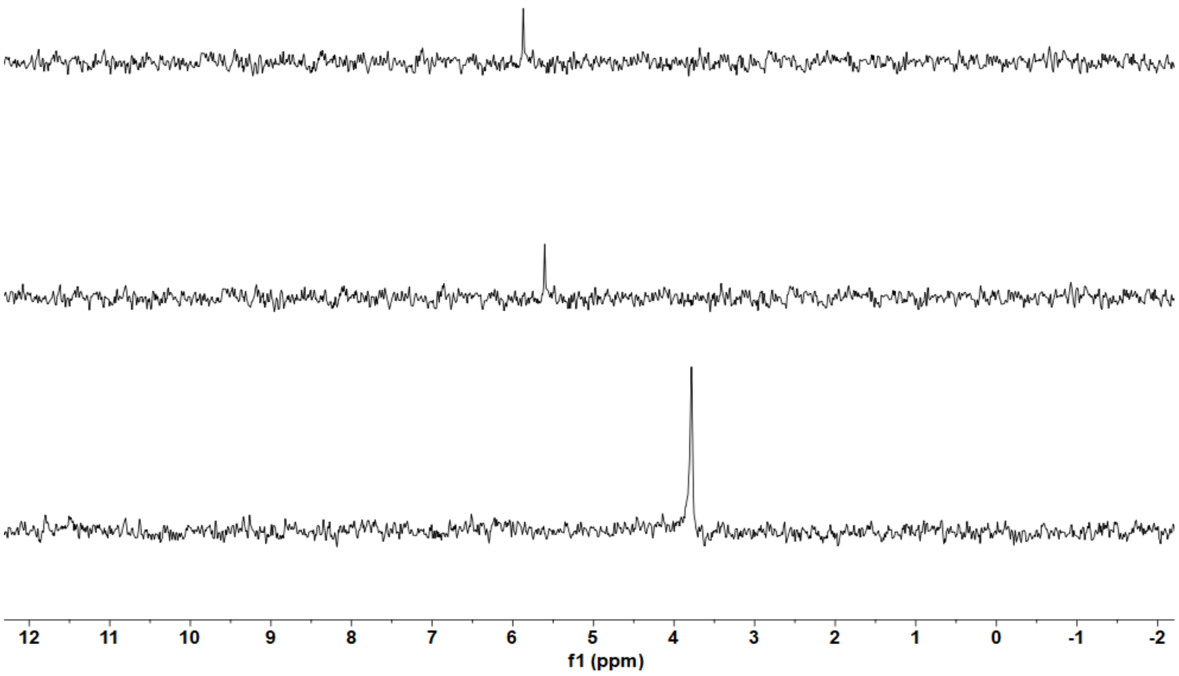

Supplementary Figure 3. Stacked ^{29}Si NMR spectra of **1*** in C_6D_6 (top, 5.87 ppm), C_6D_6 and D_8-THF (50:50, middle, 5.60 ppm), and D_8-THF (bottom, 3.78 ppm).

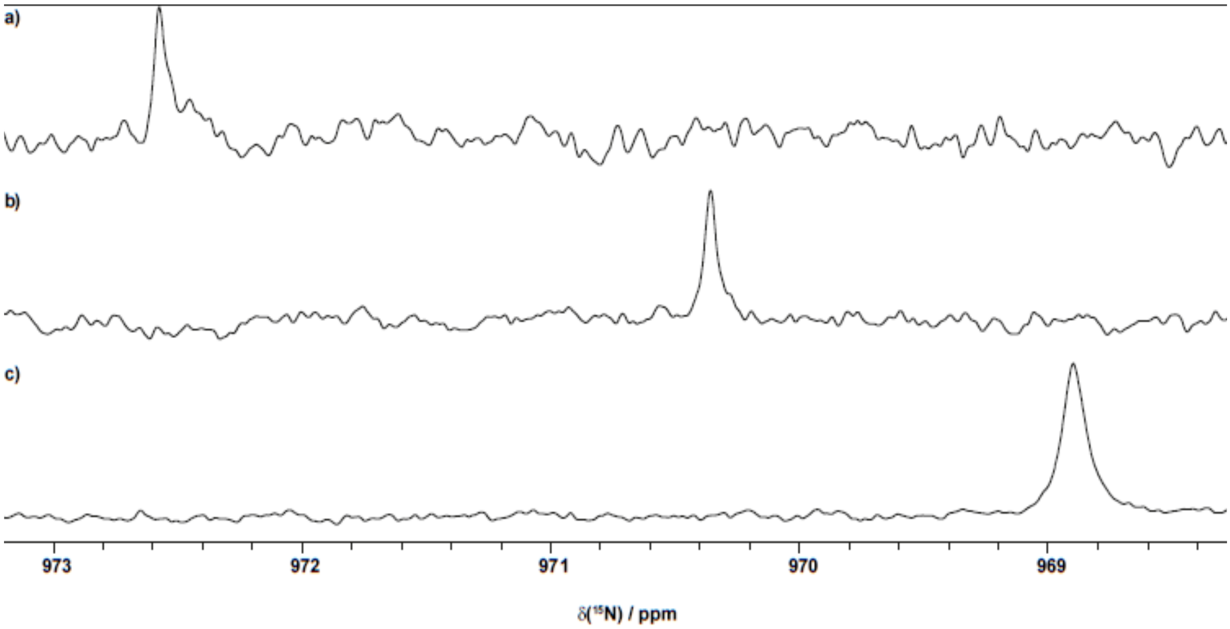

Supplementary Figure 4. Stacked solution ^{15}N NMR spectra of **1*** in C_6D_6 (top, 972.6 ppm), C_6D_6 and D_8-THF (50:50, middle, 970.4 ppm), and D_8-THF (bottom, 968.9 ppm).

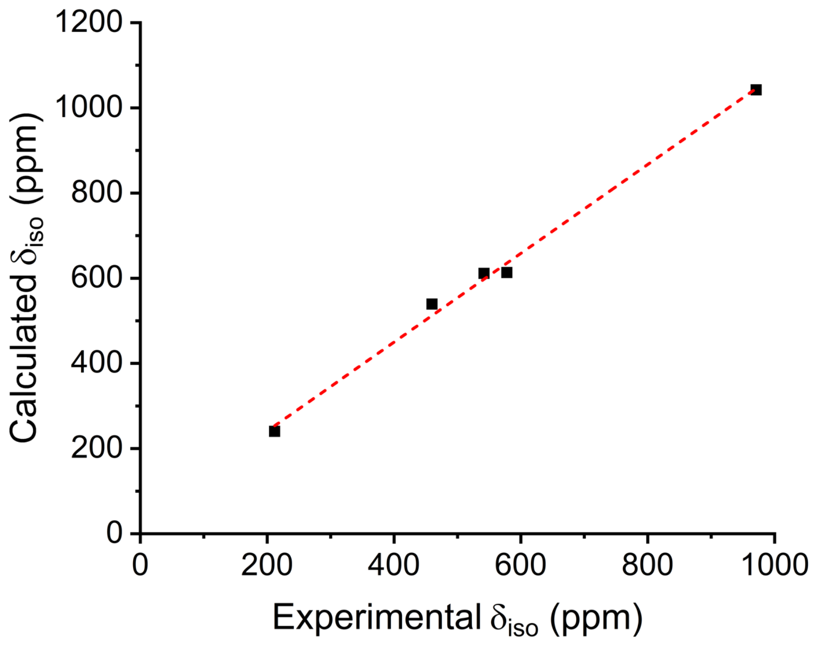

Supplementary Figure 5. Empirical scaling for solution ^{15}N data. Data points left to right, $[Mo(NBF_3)\{N\text{-}(Bu^t)(C_6H_3\text{-}3,5\text{-}Me_2)\}_3]$, $[Mo(N)\{N(Bu^t)(C_6H_3\text{-}3,5\text{-}Me_2)\}_3]$, $[Ti(\mu\text{-}N)(NP)_2K(18C6)]$, $[Ti(N)\text{-}(NP)_2]^{1-}$, and **1***. Linear regression: $\delta_{iso}(exp) = (0.9539 \times \delta_{iso}(calc)) - 28.393$, $R^2 = 0.9951$.

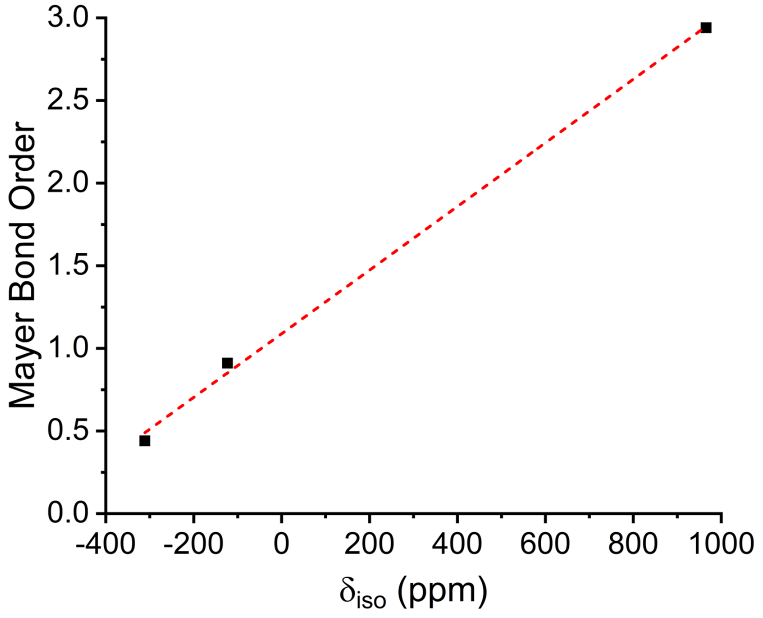

Supplementary Figure 6. Left to right, computed N_{amine}, N_{amide}, and $N_{nitride}$ δ_{iso} values for **1*** plotted vs DFT Mayer bond order. Linear regression: Mayer Bond Order = $(0.0019 \times \delta_{iso}(calc)) + 1.0885$, $R^2 = 0.9983$.

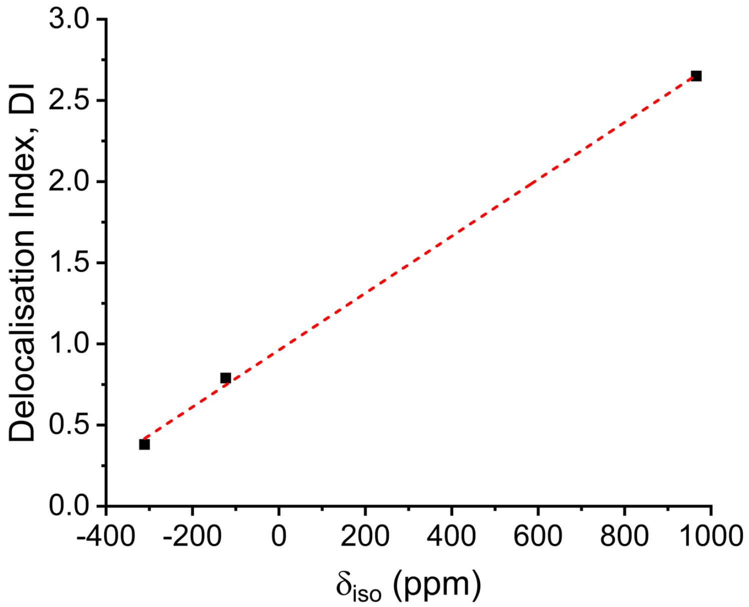

Supplementary Figure 7. Left to right, computed N_{amine}, N_{amide}, and $N_{nitride}$ δ_{iso} values for **1*** plotted vs QTAIM Delocalisation Index. Linear regression: DI = $(0.0018 \times \delta_{iso}(calc)) + 0.9608$, $R^2 = 0.9988$.

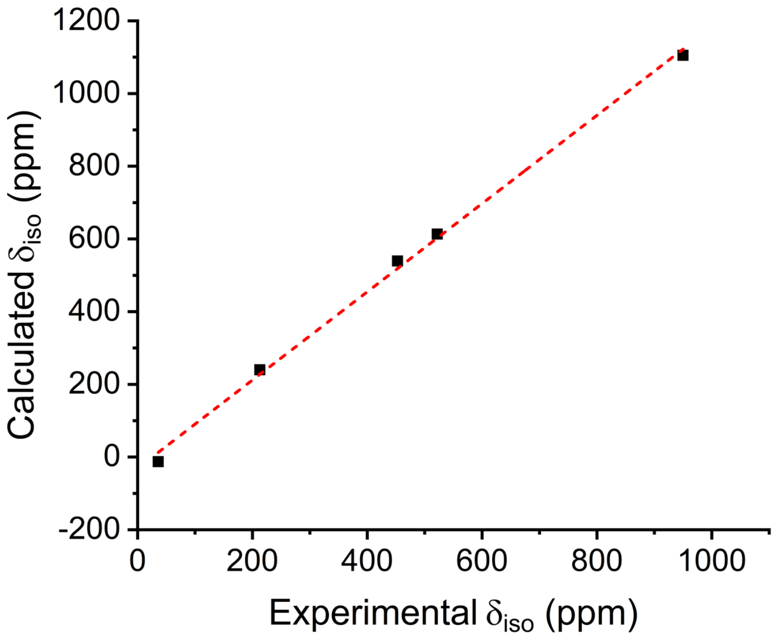

Supplementary Figure 8. Empirical scaling for solid-state ^{15}N data. Data points left to right, $[Ti(NH)(L^{ButDipp})(NTol_2)]$ $[Mo(NBF_3)\{N\text{-}(Bu^t)(C_6H_3\text{-}3,5\text{-}Me_2)\}_3]$, $[Mo(N)\{N(Bu^t)(C_6H_3\text{-}3,5\text{-}Me_2)\}_3]$, $[Ti(N)\text{-}(NP)_2]^{1-}$, and **1***. Linear regression: $\delta_{iso}(exp) = (0.8221 \times \delta_{iso}(calc)) + 26.4$, $R^2 = 0.9977$.

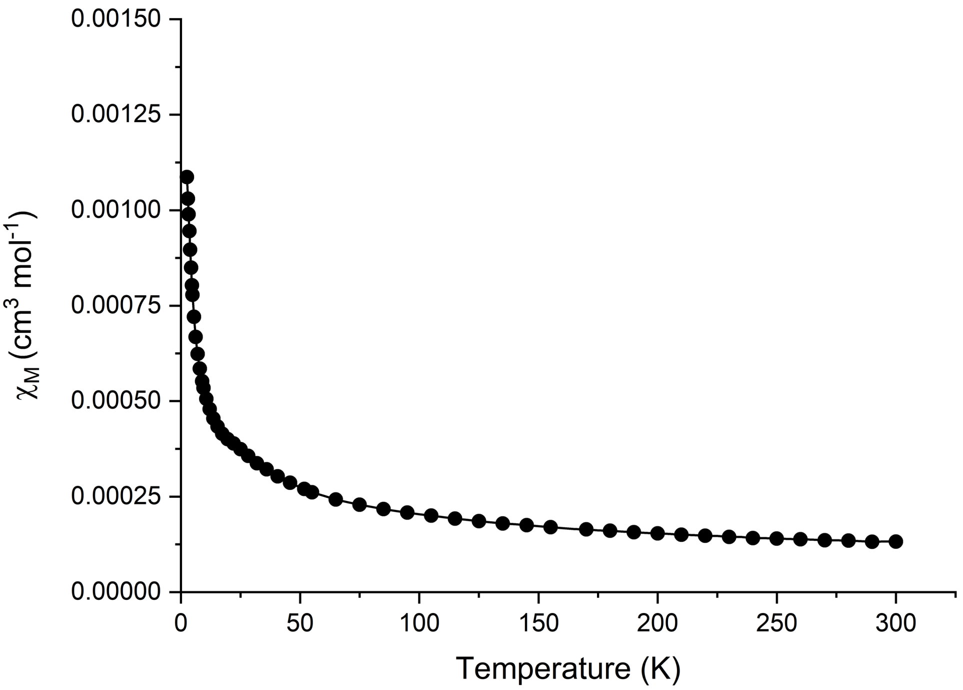

Supplementary Figure 9. Magnetic susceptibility χ vs T for **1*** over the temperature range 2-300 K.

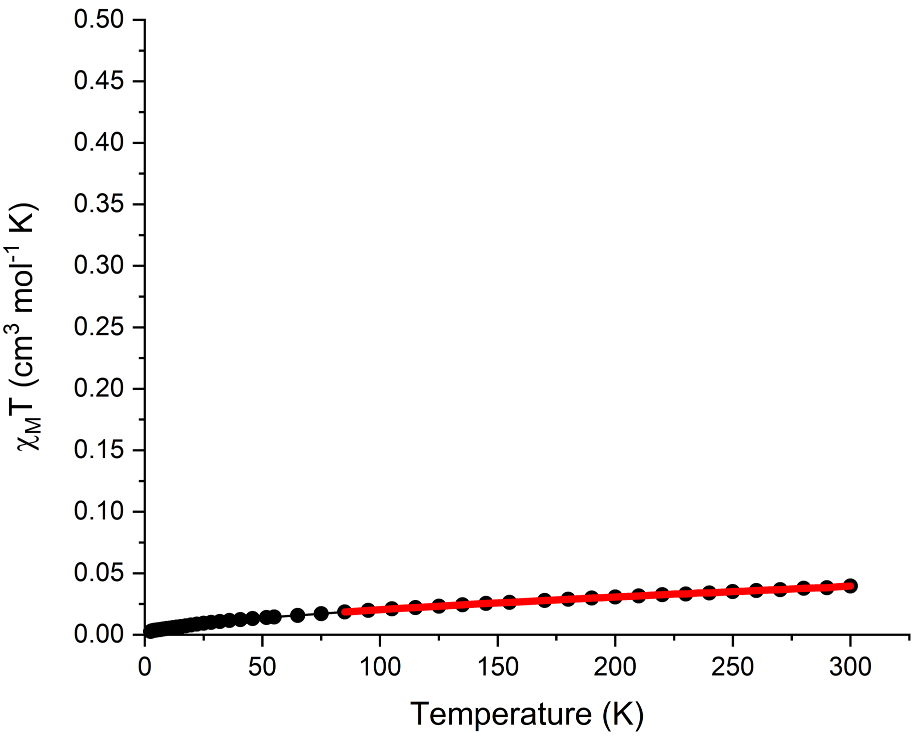

Supplementary Figure 10. Magnetic susceptibility χT vs T for **1*** over the temperature range 2-300 K. Linear regression over the range 85-300 K gives a TIP of 9.527×10^{-5} cm^3 mol^{-1} K with $R^2 = 0.997$.

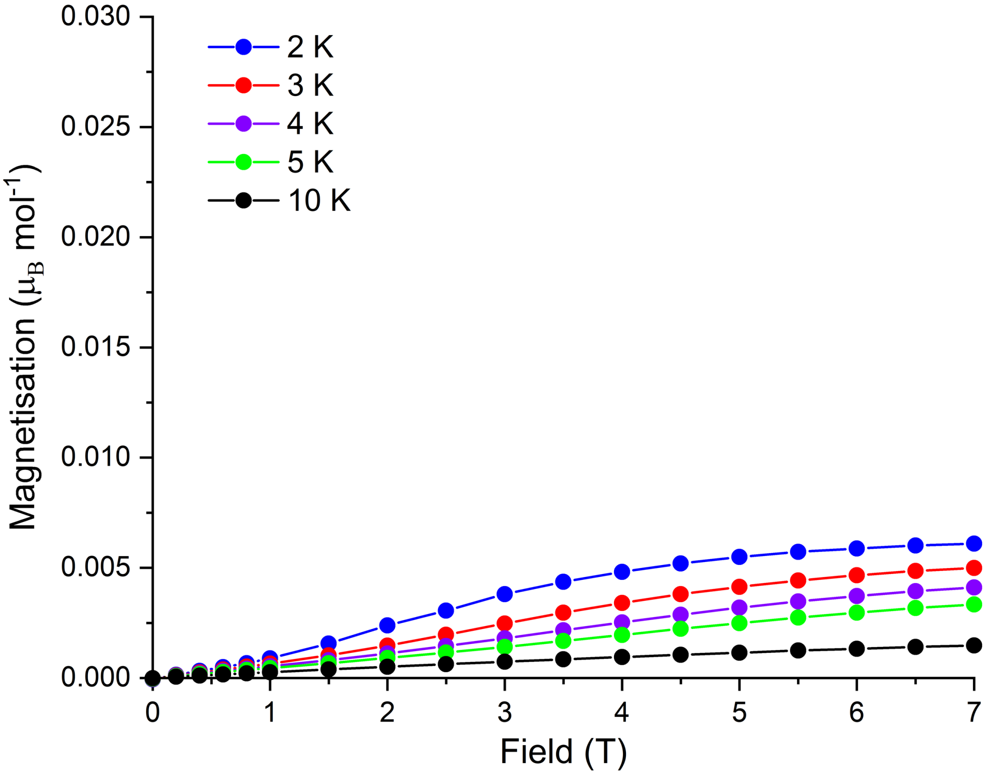

Supplementary Figure 11. Magnetisation vs Field data for **1*** at 2, 3, 4, 5, and 10 K.

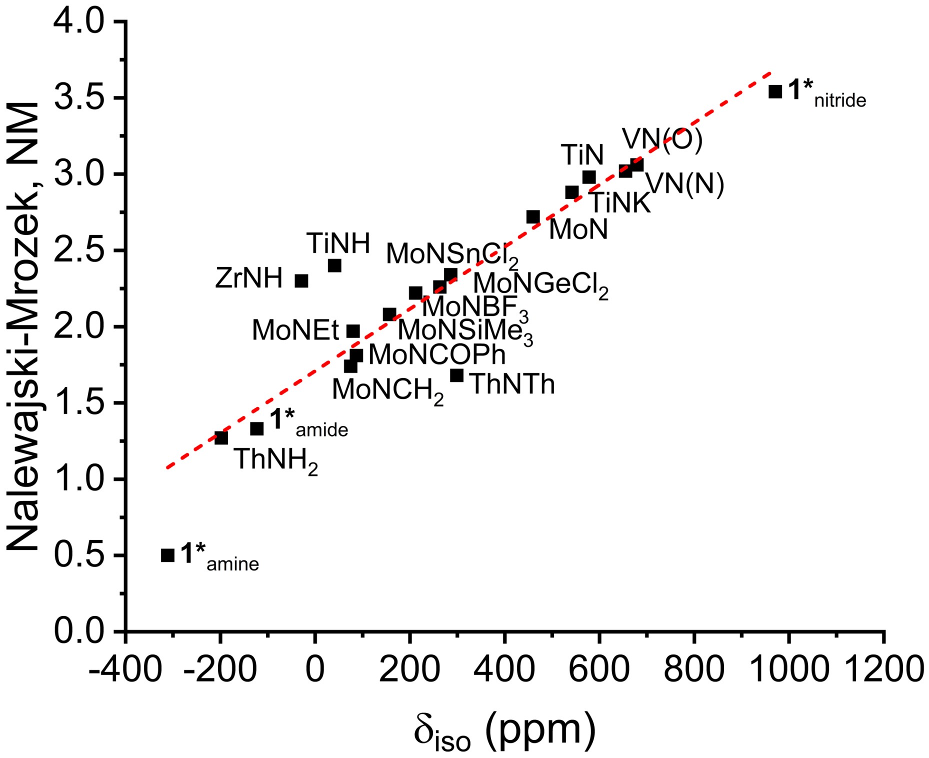

Supplementary Figure 12. Correlation of experimental ^{15}N δ_{iso} data with computed NM Bond Orders. Linear regression: NM = (0.0020 × δ_{iso}(exp)) + 1.7099, R^2 = 0.8341.

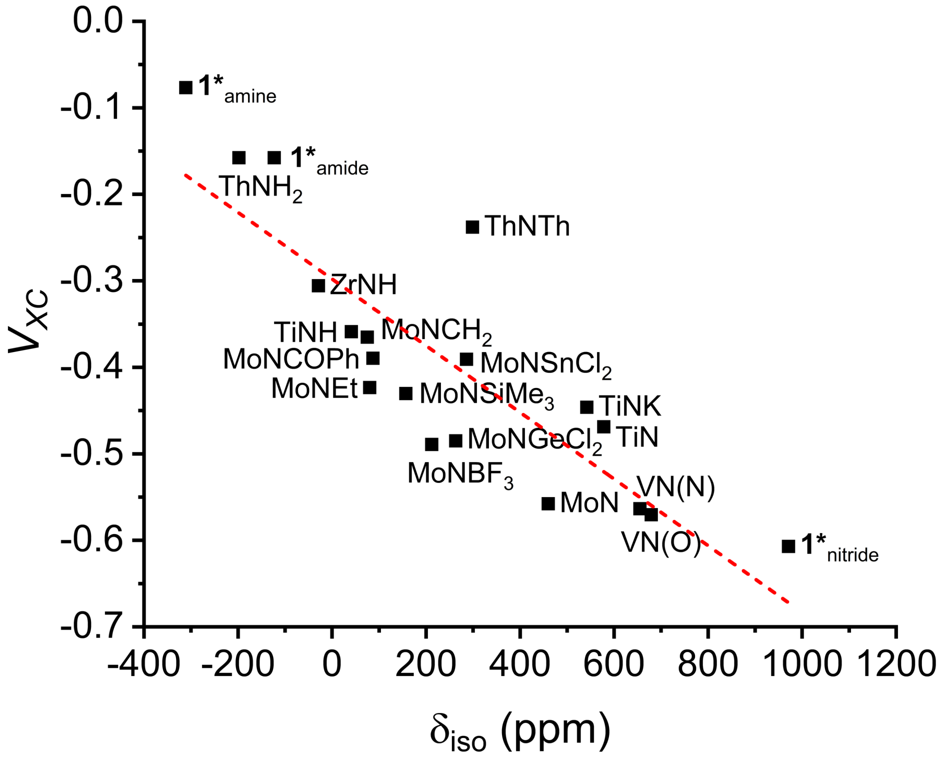

Supplementary Figure 13. Correlation of experimental [15]N δ_{iso} data with computed V_{XC}. Linear regression: $V_{XC} = (-0.0004 \times \delta_{iso}(\text{exp})) - 0.2979$, $R^2 = 0.7240$.

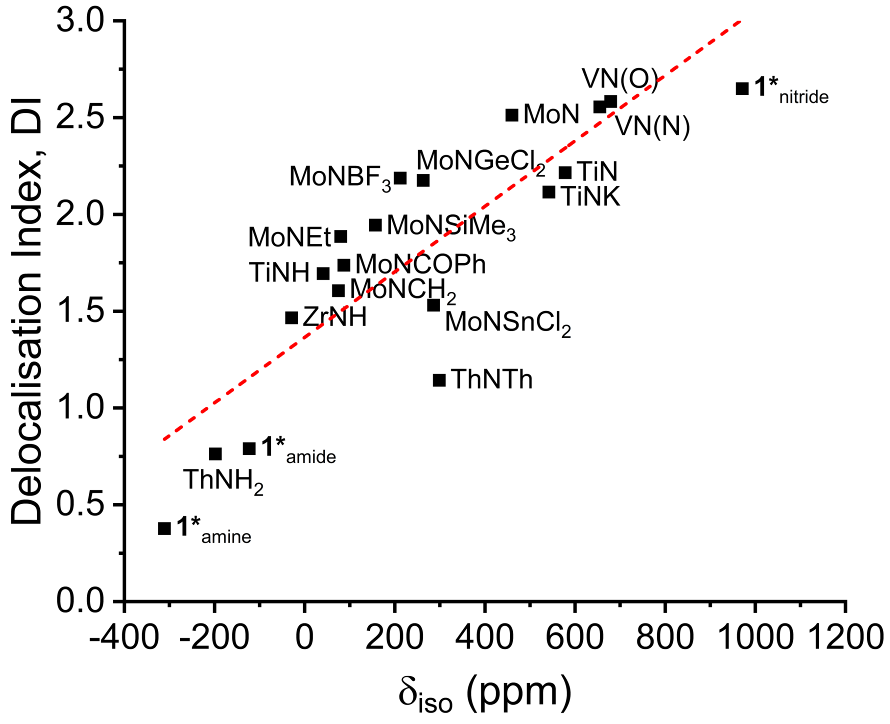

Supplementary Figure 14. Correlation of experimental [15]N δ_{iso} data with computed DI. Linear regression: $V_{XC} = (0.0017 \times \delta_{iso}(\text{exp})) + 1.3651$, $R^2 = 0.7282$.

Supplementary Tables

Supplementary Table 1. Final coordinates and energy (eV, scalar calculations) from single point energy calculations on geometry optimized structures reported in this study.

**1***	**MoN**
−724.62943660	−460.21089750
C 6.250351 -1.831846 -0.035767	Mo -0.000890 0.001525 -1.018743
C 5.449336 0.545550 -0.401190	N 0.838679 -1.774181 -0.563373
C 5.092638 -0.947806 -0.545716	N -0.001578 0.003489 -2.695711
C 3.291493 -3.805108 -1.533160	N 1.117453 1.615041 -0.560598
C 2.439627 -0.294979 -2.144150	N -1.957769 0.161492 -0.560479
C 3.126652 -3.322767 -0.079085	C 1.378423 -4.230785 -0.676658
C 1.319911 0.450743 -2.866904	C 2.193053 -2.674170 -2.484771
C 3.953565 -2.067457 2.952238	C 3.550131 -1.602208 2.038102
C 1.490388 4.822014 -1.811102	C 1.055413 -2.959728 -1.486940
C 2.455446 3.479283 0.103963	C 2.906419 -1.590143 0.799417
C 3.382449 0.357613 2.507182	C 2.816463 -1.804308 3.214421
C 3.149573 -1.098228 2.059153	C -0.249451 -3.217314 -2.260315
C 1.794941 -3.827157 0.505228	C 1.513941 -1.797169 0.704606
C -0.216007 -1.480256 -2.820348	C 1.436203 -2.002927 3.133016
C 1.296251 4.385659 -0.346207	C 0.791695 -2.008305 1.891164
C -1.122403 0.812646 -2.861951	C 2.910220 1.396603 -2.258986
C -1.012615 2.175927 -2.183452	C 2.036008 2.396701 -1.482629
C -1.421785 -2.054807 -2.079569	C 2.975976 3.309927 -0.670954
C -1.773160 4.833484 -0.579041	C 1.345709 1.684758 1.893489
C -1.620539 6.262316 -0.015539	C -3.157294 -2.276419 2.040767
C 0.128735 4.449711 2.812564	C -2.826505 -1.724128 0.801982
C -2.327959 -5.027308 -0.074405	C 1.220656 3.242080 -2.478295
C -0.449653 3.296123 1.965348	C 0.800148 2.207598 0.709035
C -4.057794 -4.150617 -1.691045	C -2.966666 -1.539651 3.217008
C -3.429004 -3.968040 -0.290995	C 1.020277 2.237266 3.136948
C -1.520506 -2.902312 2.652563	C -2.312923 -0.413394 0.707329
C -3.226231 4.343919 -0.416844	C -3.419774 -0.558087 -2.479197
C -1.816966 2.859498 2.527102	C -2.451128 -0.243971 3.135619
C -2.632369 -2.001529 2.078997	C -2.135446 0.317773 1.893843
C -4.350478 -0.975277 -0.371306	C -3.094462 0.568498 -1.480968
C -4.122736 0.462691 0.129431	C -0.076612 3.308718 0.807466
C -5.794180 -1.421848 -0.060118	C 0.157322 3.332345 3.221908
C -3.919562 -2.171742 2.912625	C -2.664631 1.827264 -2.254096
H 7.190746 -1.576464 -0.554302	C -0.387285 3.868750 2.047837
H 6.341117 0.797734 -0.999487	C -4.354846 0.924215 -0.667810
H 6.065192 -2.903438 -0.203319	H 1.443673 -5.084151 -1.367822
H 6.428260 -1.690658 1.041585	H 2.309981 -3.517605 -3.182806
H 4.994526 -1.154990 -1.628793	H 2.333850 -4.155682 -0.139263
H 4.236492 -3.470995 -1.986654	H 3.151060 -2.535306 -1.965941
H 4.632904 1.208411 -0.721227	H 4.628246 -1.441992 2.087392
H 5.677346 0.800939 0.643817	H 0.587545 -4.445168 0.057005
H 3.271138 -4.906914 -1.588436	H 3.478178 -1.404394 -0.108976
H 3.355272 0.307686 -2.275592	H -0.133961 -4.103536 -2.901866
H 2.642333 -1.248725 -2.665915	H 1.968367 -1.771171 -3.067376
H 3.942299 -3.782847 0.510341	H 3.317987 -1.804846 4.183311
H 2.469727 -3.442215 -2.170980	H 3.485040 0.772220 -1.560845
H 1.436393 0.379568 -3.965571	H -1.075799 -3.403219 -1.560119
H 5.038936 -1.909659 2.855777	H -0.508744 -2.362496 -2.894453
H 2.446475 5.358276 -1.937342	H 3.619138 1.940872 -2.900461
H 3.434493 3.938778 -0.111635	H 0.849429 -2.158143 4.039439
H 4.450287 0.623286 2.487394	H 2.298452 0.746290 -2.893736

H	1.355012	1.510685	-2.586483
H	1.522729	3.956000	-2.491246
H	3.752123	-3.123573	2.723136
H	2.431867	2.507056	-0.414127
H	0.684761	-2.030067	-2.519805
H	-0.333515	-1.582077	-3.916438
H	1.684868	-4.915900	0.368209
H	0.690844	5.490059	-2.163486
H	2.845821	1.075819	1.874487
H	3.697798	-1.909197	4.013346
H	2.424155	3.272132	1.182132
H	0.930393	-3.353151	0.011804
H	3.024193	0.503030	3.538908
H	-1.150043	0.914916	-3.963753
H	1.709719	-3.624674	1.581515
H	-0.300145	2.807121	-2.744484
H	1.336190	5.303024	0.271018
H	2.077363	-1.300286	2.241755
H	-1.352560	-3.153356	-2.166539
H	-2.050414	0.322452	-2.540094
H	-0.614855	6.675049	-0.183688
H	-2.349865	-1.777573	-2.616983
H	1.123769	4.771832	2.472268
H	-1.441918	-4.826832	-0.696635
H	-1.571890	4.898136	-1.666124
H	-1.993061	2.670510	-2.302617
H	0.223496	2.427995	2.101298
H	-3.291275	-4.149171	-2.480612
H	-2.696353	-6.031500	-0.346963
H	0.230273	4.134009	3.865352
H	-2.341577	6.949115	-0.491584
H	-0.603149	-2.876005	2.051663
H	-1.987739	-5.071597	0.967835
H	-1.814209	6.292026	1.068100
H	-0.527640	5.334766	2.802209
H	-4.788963	-3.369441	-1.944035
H	-3.367656	3.314053	-0.773201
H	-4.577914	-5.121577	-1.754414
H	-3.124086	0.847299	-0.130489
H	-1.252450	-2.572524	3.668806
H	-3.923221	4.991282	-0.975578
H	-1.850629	-3.949841	2.725318
H	-4.253315	-0.954970	-1.475211
H	-2.258038	2.033632	1.954370
H	-2.283481	-0.961347	2.219000
H	-4.230985	-4.178683	0.445005
H	-3.539533	4.363173	0.637142
H	-1.713324	2.513051	3.568091
H	-2.536544	3.692844	2.526829
H	-4.859244	1.158744	-0.305348
H	-4.227073	0.529125	1.222769
H	-6.009712	-2.441486	-0.408570
H	-4.380048	-3.161836	2.761824
H	-6.521116	-0.748011	-0.544587
H	-3.691912	-2.083302	3.988332
H	-4.678605	-1.413296	2.677791
H	-6.002864	-1.395226	1.019195
N	2.108674	-0.504598	-0.723348
N	-0.003507	-0.063640	-2.442513
N	-0.626146	2.034061	-0.767911
H	-0.284223	-2.176251	1.829822
H	3.557674	2.730549	0.060841
H	2.029415	0.837422	1.829119
H	3.681606	3.794884	-1.361635
H	-3.555492	-3.290968	2.090422
H	-2.949986	-2.312173	-0.106648
H	1.894133	3.765629	-3.174444
H	-2.527394	-0.816201	-3.064356
H	1.449600	1.804848	4.041737
H	0.550070	2.598592	-3.062790
H	2.433345	4.098468	-0.131449
H	-3.216749	-1.974448	4.185825
H	-3.779253	-1.456876	-1.960333
H	0.622234	4.001721	-1.957535
H	-4.209657	-0.234766	-3.174842
H	-1.795183	1.623477	-2.888679
H	-2.292500	0.341945	4.041734
H	-1.744883	1.334259	1.832453
H	-0.524790	3.712531	-0.099564
H	-0.091889	3.764795	4.191914
H	-4.766913	0.058287	-0.131399
H	-2.411208	2.635543	-1.553926
H	-3.489832	2.171180	-2.895236
H	-4.143676	1.714941	0.066846
H	-5.127420	1.295665	-1.357269
H	-1.065783	4.721611	2.100228

N -1.448650 -1.612480 -0.675986	
N 0.028117 0.009402 1.871474	
Si 3.362483 -1.422499 0.176999	
Si -0.425353 3.608418 0.068567	
Si -2.923104 -2.161081 0.184874	
U 0.013493 -0.011149 0.092117	
**MoNBF$_3$**	**MoNGeCl$_2$**
−581.80429903	−568.59887021
C 4.434707 0.493533 -2.321109	C -0.096270 -3.656798 2.535493
C 1.801121 5.052791 -1.548073	C -2.337650 -4.661003 -2.457894
C 3.159250 -0.309390 -2.429387	C 1.713340 -3.191673 0.900962
C 3.182749 -1.644530 -2.841978	C 0.835761 -2.556930 1.988622
C 3.006311 3.496476 0.043304	C 1.715512 -2.012721 3.128884
C 1.805185 3.916004 -0.551339	C -2.279380 -3.151282 -2.401122
C 4.342269 2.017689 1.602526	C -1.083108 -2.496111 -2.085560
C -0.365514 1.338777 -4.136095	C 2.175295 -1.538022 -2.955083
C 1.921190 0.280171 -2.118214	C -2.421431 -1.698098 1.262234
C 3.038671 2.440702 0.963561	C -3.418767 -2.375530 -2.668074
C 2.003288 -2.403815 -2.946009	C -4.911610 -2.106434 1.084320
C 2.063854 -3.852174 -3.374387	C -1.291830 -1.212657 1.929486
C 4.388721 -2.348969 0.998564	C -3.707893 -1.555200 1.812172
C 0.729346 -0.446494 -2.222216	C -1.460645 -0.555149 3.165071
C 0.620966 3.256601 -0.204717	C -3.842026 -0.911413 3.045303
C 0.786683 -1.793612 -2.631839	C -1.013330 -1.092058 -2.049697
C -1.327806 0.686190 -3.122732	C -2.726655 -0.402976 3.735967
C 1.836043 1.798043 1.294461	C 1.198901 -0.352401 -2.997013
C -2.082386 -0.478370 -3.788724	C 0.415052 -0.380341 -4.324018
C 0.619865 2.201367 0.725516	C -3.382777 -0.975752 -2.622095
C -2.330950 1.756102 -2.670341	C -2.908700 0.295611 5.064058
C 3.073447 -2.015102 1.662410	C -2.169932 -0.344303 -2.307672
C 1.882235 -2.022848 0.916303	C 1.965858 0.975768 -2.933685
C 3.010645 -1.703577 3.024247	C -4.614143 -0.153223 -2.922815
C 0.650526 -1.738820 1.518714	C 1.604823 2.100086 2.622379
C -0.579048 3.085147 3.100406	C -2.393100 2.070772 0.649770
C 1.790450 -1.400307 3.655057	C 0.828318 2.829476 1.516153
C -2.425698 3.273798 1.396281	C -1.143424 2.298587 0.056432
C -1.476025 2.303796 2.119540	C -4.904016 2.354516 0.747564
C 0.620948 -1.423848 2.891261	C 1.799824 3.617958 0.621713
C -0.380763 -4.270091 0.669133	C -3.567311 2.588781 0.082475
C 1.755130 -1.050978 5.124885	C -1.078186 3.038246 -1.137444
C -1.354438 -3.081703 0.809340	C -0.148563 3.804330 2.205145
C -2.347365 -3.150515 -0.358819	C -3.469532 3.336608 -1.098190
C -2.290997 1.289248 2.935158	C -2.234125 3.568934 -1.723181
C -2.121100 -3.182151 2.140394	C -2.149357 4.372862 -3.000824
H 4.424560 1.350695 -3.009638	H 0.524459 -4.482777 2.912346
H 5.315104 -0.118094 -2.555839	H 2.282309 -4.029334 1.329401
H 2.393069 4.803143 -2.441087	H -2.090331 -5.109947 -1.484965
H 2.241695 5.961413 -1.113574	H -1.619060 -5.058703 -3.188747
H 3.937854 4.007078 -0.219128	H -0.747560 -4.054242 1.744022
H 4.555772 0.900031 -1.307363	H -3.337160 -5.010354 -2.745824
H 4.142241 -2.110676 -3.084735	H 1.086907 -3.584646 0.088330
H 5.097590 1.762149 0.844260	H -0.730182 -3.302550 3.359833
H 0.783564 5.295741 -1.876846	H 2.338228 -2.818101 3.546394
H 2.534311 -3.955024 -4.362533	H 2.434556 -2.484857 0.477427
H 4.765039 2.829060 2.213706	H -0.185053 -3.073811 -1.866588
H 0.367210 0.626384 -4.538369	H -4.843432 -3.199009 0.977943
H -0.954617 1.733015 -4.977071	H 1.639174 -2.493188 -3.040967
H 0.179725 2.176707 -3.677543	H 1.101181 -1.606788 3.943943
H 1.876468 1.322592 -1.797012	H 2.382597 -1.220791 2.761941
H 4.577338 -1.686029 0.142479	H -2.288628 -2.207777 0.306263

H 2.659966 -4.450618 -2.669700	H 2.879186 -1.472161 -3.798225
H 4.200999 1.141882 2.246695	H -4.357041 -2.877367 -2.920108
H 5.227506 -2.255532 1.700090	H 2.764261 -1.537782 -2.029039
H 4.385510 -3.378809 0.612110	H -5.842983 -1.877428 1.617589
H 1.063051 -4.296710 -3.429033	H -0.122994 -1.325982 -4.476416
H -0.319833 3.553898 -0.666593	H -4.980036 -1.691686 0.068812
H -1.385672 -1.232087 -4.179725	H -3.462617 -0.338386 5.771211
H -2.675275 -0.102708 -4.636225	H -0.584364 -0.158871 3.677851
H -2.823460 2.182745 -3.555529	H -4.837872 -0.802401 3.484588
H -0.137031 -2.368431 -2.684747	H 1.135045 -0.262800 -5.146992
H -1.817562 2.571147 -2.141926	H -1.944737 0.549593 5.521548
H 1.906385 -2.261097 -0.148321	H -5.479904 -0.790950 -3.141859
H 1.833478 0.976921 2.013146	H 2.646320 1.047856 -3.794210
H 3.933130 -1.694010 3.611924	H -0.308055 0.445790 -4.383500
H -2.769737 -0.950548 -3.074296	H 2.352728 1.410906 2.214060
H -0.006336 3.880248 2.603278	H 2.576693 1.061292 -2.028645
H -3.111116 1.352917 -2.018326	H -3.482697 1.227462 4.949458
H -1.863826 4.042012 0.848521	H 0.918781 1.532020 3.265786
H 0.172380 -4.213603 -0.279075	H -4.868666 0.498599 -2.074983
H 0.127410 2.417101 3.613221	H -2.109073 0.745187 -2.277070
H 2.360646 -0.157110 5.336751	H -4.449185 0.502305 -3.790213
H -1.220712 3.552843 3.861213	H -2.438871 1.489853 1.572958
H -1.831816 -3.008137 -1.319156	H 1.267037 1.823195 -2.978193
H -3.083528 2.735707 0.701480	H 2.131904 2.835627 3.246571
H 0.344688 -4.318562 1.492740	H -5.095138 1.280318 0.880230
H -3.060450 3.789068 2.133199	H 2.511624 2.949333 0.119403
H 2.161678 -1.868640 5.737454	H -0.868879 3.267971 2.839502
H -0.962359 -5.203247 0.671430	H -4.933391 2.810287 1.748116
H -0.335142 -1.181559 3.353521	H -0.107739 3.189181 -1.610059
H 0.731737 -0.851663 5.465295	H -5.725677 2.782638 0.159512
H -3.146581 -2.409543 -0.280572	H 2.375088 4.332518 1.229957
H -1.620964 0.602316 3.470610	H 1.254310 4.193221 -0.138140
H -2.816445 -4.144473 -0.371768	H 0.432575 4.484133 2.845512
H -2.978625 0.706135 2.314590	H -0.708550 4.413562 1.482793
H -2.892902 1.825900 3.682366	H -1.764748 3.764898 -3.832664
H -1.433998 -3.195016 2.996748	H -4.378129 3.749392 -1.545339
H -2.821555 -2.342242 2.246643	H -3.133300 4.760143 -3.293806
H -2.702285 -4.116178 2.164079	H -1.470437 5.229906 -2.887221
B -4.423202 -0.037535 -0.008115	N 0.022841 -1.422955 1.373744
N -0.536909 0.182877 -1.924064	N 2.354103 -0.021850 0.183222
N -0.611186 1.555467 1.109194	N 0.239509 -0.423170 -1.805470
N -0.570577 -1.778801 0.748117	N 0.058670 1.809172 0.686524
N -2.806251 -0.017844 -0.039158	Cl 5.079639 -1.777693 -0.444852
F -4.851661 1.283319 -0.187476	Cl 4.980417 1.741633 -0.774835
F -4.838531 -0.869322 -1.055072	Mo 0.641333 -0.011720 0.101704
F -4.790078 -0.545459 1.245039	Ge 4.393287 0.086802 0.715889
Mo -1.096772 -0.015820 -0.020305	
**$MoNSnCl_2$**	**$MoNSiMe_3$**
−568.12445760	−618.73600095
C -0.267262 -3.572418 2.662085	C -1.830373 4.861817 -2.076322
C -2.635060 -4.729928 -2.214475	C 0.095737 1.325963 -4.020539
C 1.523792 -3.161148 0.992345	C 2.157040 1.682007 -2.678860
C 0.647495 -2.488267 2.058794	C 1.829476 -0.499184 -3.880232
C 1.532913 -1.888106 3.165668	C 1.125558 0.630146 -3.108095
C -2.563063 -3.220051 -2.230208	C -1.907620 3.811503 -0.992755
C -1.353928 -2.563153 -1.973105	C -3.139553 3.435504 -0.435699
C 1.922641 -1.679146 -2.891834	C -0.745414 3.193000 -0.514685
C -2.630326 -1.669843 1.337866	C -4.559168 -0.027073 -2.410654
C -3.702217 -2.445683 -2.502532	C -2.047121 -0.040037 -2.113898
C -5.128611 -2.043844 1.192618	C -3.225863 -0.736841 -2.422331

C	-1.490420	-1.158402	1.967443	C	5.156446	-0.755675	-1.616891
C	-3.910879	-1.481495	1.887886	C	-0.802604	-0.682283	-2.162691
C	-1.642647	-0.433272	3.166636	C	-3.135619	-2.094384	-2.757604
C	-4.027939	-0.770131	3.085066	C	5.152496	1.785825	0.158433
C	-1.270179	-1.159810	-2.001338	C	-0.741273	-2.041604	-2.509539
C	-2.901827	-0.236831	3.738418	C	-1.903631	-2.764819	-2.805481
C	0.944835	-0.498685	-3.004358	C	-3.229880	2.466706	0.572742
C	0.154587	-0.610982	-4.322960	C	-1.826131	-4.228673	-3.172035
C	-3.652794	-1.045863	-2.519205	C	1.819583	3.613126	1.511686
C	-3.065742	0.542536	5.023413	C	-0.806885	2.212872	0.489171
C	-2.425981	-0.413172	-2.265791	C	-4.562933	2.102878	1.182828
C	1.712353	0.830724	-3.029316	C	-2.051444	1.849972	1.021193
C	-4.887246	-0.225445	-2.812729	C	1.118442	2.376766	2.101088
C	1.415343	2.229969	2.498170	C	0.087305	2.815076	3.160460
C	-2.615444	2.086244	0.613208	C	2.157580	-3.157965	-0.115142
C	0.630647	2.895018	1.357027	C	5.152722	-1.023188	1.473039
C	-1.379932	2.288852	-0.017179	C	2.151451	1.480931	2.796983
C	-5.127754	2.349582	0.741266	C	-2.049528	-1.810126	1.087434
C	1.596865	3.610797	0.397536	C	-4.561353	-2.075566	1.219695
C	-3.804647	2.566119	0.043725	C	-0.806077	-1.531885	1.670569
C	-1.343887	2.965761	-1.248772	C	1.122802	-3.006078	1.007681
C	-0.322276	3.925118	1.996250	C	-3.229544	-1.730565	1.843228
C	-3.735608	3.253147	-1.175243	C	0.093826	-4.144290	0.855900
C	-2.514780	3.459776	-1.836553	C	-0.747069	-1.154715	3.022089
C	-2.461559	4.192624	-3.157719	C	-3.141657	-1.344064	3.187236
H	0.365609	-4.367795	3.083108	C	1.823396	-3.112475	2.373344
H	2.121595	-3.959342	1.456121	C	-1.910895	-1.051233	3.794504
H	-2.347171	-5.135149	-1.233618	C	-1.837477	-0.639944	5.246617
H	-1.952603	-5.167250	-2.957046	H	0.630067	1.786460	-4.863931
H	-0.912346	-4.020544	1.893223	H	-1.378971	4.459626	-2.994776
H	-3.648667	-5.083118	-2.440938	H	-2.825276	5.245046	-2.332338
H	0.897111	-3.613611	0.211548	H	-1.213456	5.714605	-1.759971
H	-0.907107	-3.183336	3.467023	H	2.600279	2.136568	-3.575011
H	2.165064	-2.669091	3.614698	H	2.373905	-0.078189	-4.738206
H	2.218258	-2.459330	0.518320	H	-0.641459	0.622822	-4.428099
H	-0.456799	-3.139454	-1.746529	H	-0.438671	2.117173	-3.477216
H	-5.063550	-3.137348	1.099539	H	1.686706	2.481722	-2.092102
H	1.385496	-2.637537	-2.892963	H	1.107023	-1.227262	-4.269880
H	0.922362	-1.448235	3.966423	H	-4.607755	0.729507	-3.207132
H	2.188481	-1.107675	2.756711	H	-4.052403	3.915845	-0.796296
H	-2.511546	-2.228671	0.407978	H	2.963716	1.244409	-2.086362
H	2.612022	-1.680239	-3.749467	H	2.550850	-1.021748	-3.237576
H	-4.651565	-2.948332	-2.705988	H	0.223320	3.470500	-0.929050
H	2.528433	-1.611811	-1.979201	H	4.858826	-0.153754	-2.487054
H	-6.049254	-1.805072	1.739705	H	4.777063	2.418972	-0.657749
H	-0.397347	-1.557890	-4.404289	H	-2.090320	1.016778	-1.846325
H	-5.216425	-1.642932	0.172789	H	-5.388443	-0.728035	-2.565599
H	-3.585427	-0.055019	5.785923	H	6.257729	-0.802891	-1.609172
H	-0.758569	-0.011824	3.644736	H	-1.208515	-4.381911	-4.068198
H	-5.019425	-0.621863	3.522214	H	-4.721832	0.500214	-1.460723
H	0.870794	-0.563013	-5.156497	H	1.095827	4.313521	1.076154
H	-2.096742	0.847232	5.437178	H	6.253744	1.805243	0.115730
H	-5.757210	-0.864350	-3.009912	H	-4.048875	-2.646736	-2.992040
H	2.402988	0.840585	-3.884826	H	4.780969	-1.778619	-1.760290
H	-0.559002	0.218439	-4.437416	H	2.542866	3.321391	0.738185
H	2.123660	1.482361	2.123983	H	0.227430	-2.539289	-2.542489
H	2.313415	0.981343	-2.126206	H	-5.392292	2.589810	0.655544
H	-3.663889	1.452238	4.864086	H	-2.820891	-4.642278	-3.376142
H	0.730617	1.738726	3.203777	H	4.851717	2.239339	1.113362
H	-5.127859	0.435919	-1.968099	H	2.361512	4.146032	2.306792

H -2.355718 0.675978 -2.283956<br>H -4.735417 0.420671 -3.689145<br>H -2.638361 1.552847 1.565451<br>H 1.015760 1.673962 -3.141448<br>H 1.984926 2.993699 3.046974<br>H -5.318968 1.278322 0.896231<br>H 2.290249 2.902154 -0.074650<br>H -1.027749 3.442001 2.688026<br>H -5.135625 2.822205 1.734047<br>H -0.384866 3.093153 -1.750842<br>H -5.960285 2.769118 0.162561<br>H 2.191781 4.356610 0.946393<br>H 1.045225 4.141326 -0.390006<br>H 0.279005 4.647461 2.568484<br>H -0.899365 4.482032 1.245355<br>H -2.070630 3.547445 -3.957853<br>H -4.656029 3.634886 -1.625356<br>H -3.457054 4.539939 -3.461114<br>H -1.801849 5.069973 -3.100003<br>N -0.183172 -1.391604 1.404340<br>N 2.117734 -0.012311 0.158538<br>N -0.007144 -0.493635 -1.806006<br>N -0.162992 1.833049 0.608456<br>Cl 5.019073 -1.947787 -0.604846<br>Cl 4.891184 1.806861 -1.122891<br>Mo 0.410768 -0.011124 0.082777<br>Sn 4.421544 0.115802 0.626994	H -1.375186 -4.822195 -2.363659<br>H -4.727426 1.016830 1.162552<br>H 1.690404 -3.045596 -1.102159<br>H -4.609327 2.412437 2.237058<br>H -0.652217 3.517464 2.755793<br>H -2.094752 1.089486 1.802407<br>H 2.964980 -2.427485 -0.027391<br>H -4.722774 -1.514707 0.288932<br>H 2.959542 1.189870 2.122071<br>H 6.253944 -0.994504 1.511513<br>H -2.090661 -2.104704 0.037697<br>H 0.620312 3.317000 3.980800<br>H 4.853216 -2.077294 1.388187<br>H 2.599243 -4.162117 -0.062653<br>H 2.592383 2.029751 3.639969<br>H 4.776382 -0.633308 2.429234<br>H -0.437389 -4.068298 -0.102591<br>H -0.444153 1.947303 3.574130<br>H -4.608201 -3.143272 0.960406<br>H 1.682886 0.571180 3.194306<br>H -5.392146 -1.861654 1.903031<br>H 0.628193 -5.104918 0.879529<br>H 2.543237 -2.293782 2.508111<br>H -0.646226 -4.146881 1.666137<br>H 0.220627 -0.934901 3.472064<br>H -4.055599 -1.272450 3.782053<br>H 2.368999 -4.065424 2.436859<br>H 1.099115 -3.088742 3.197282<br>H -1.386958 0.356646 5.360020<br>H -2.833815 -0.610891 5.703529<br>H -1.222034 -1.340720 5.828075<br>N 0.412737 0.061702 -1.884262<br>N 2.767689 0.000743 0.002563<br>N 0.408570 1.600458 0.995127<br>N 0.410313 -1.661929 0.888504<br>Si 4.568985 0.001520 0.004650<br>Mo 1.016704 0.000342 0.000267
**MoNCOPh**	**MoNEt**
−639.02789731<br>C 0.018006 2.630155 -3.222199<br>C -0.623200 0.436361 -4.298366<br>C -2.126939 1.525330 -2.624515<br>C -0.667463 1.266267 -3.003242<br>C 0.333264 5.540022 0.069256<br>C 4.773137 2.174419 -1.562104<br>C 3.780225 1.112847 -1.973039<br>C 2.413834 1.269111 -1.689976<br>C 4.197612 -0.034460 -2.660560<br>C 1.482284 0.314883 -2.121858<br>C 0.787687 4.225381 0.659017<br>C -5.771743 1.685059 0.368817<br>C 1.927097 -0.814475 -2.825894<br>C 3.287683 -1.012675 -3.089774<br>C -7.035262 1.086377 0.412956<br>C 3.757186 -2.252656 -3.814516<br>C -4.662306 0.936124 -0.020472<br>C -7.190060 -0.258177 0.052318<br>C -0.076927 3.124541 0.697525<br>C 2.073745 4.081153 1.204029<br>C -6.085053 -1.006506 -0.346333	−588.84782011<br>C 0.903350 0.873350 -4.128868<br>C -1.089710 2.347199 -3.657082<br>C 1.928572 5.229303 -0.451680<br>C -0.417907 0.997416 -3.344144<br>C 2.395253 -3.575847 -3.567585<br>C 3.261530 3.070573 -0.544556<br>C -1.335327 -0.147233 -3.791768<br>C 2.040382 3.743799 -0.704561<br>C 4.720748 1.002839 -0.651757<br>C 3.388296 1.698689 -0.799378<br>C 0.926324 3.007236 -1.123461<br>C 2.253606 0.982513 -1.213259<br>C 1.021760 1.629430 -1.379495<br>C 2.106189 -3.086744 -2.167286<br>C 3.107685 -3.064317 -1.183949<br>C 0.820033 -2.663103 -1.813348<br>C 2.848599 -2.637554 0.125355<br>C -4.205797 0.378477 -1.508673<br>C 0.531507 -2.227397 -0.509255<br>C 3.935169 -2.658127 1.174423<br>C -2.549783 3.042154 0.529240

C -4.807736 -0.415155 -0.373397	C 1.550952 -2.211337 0.451661
C -3.660278 -1.274142 -0.768980	C -2.298987 -3.618955 -1.128812
C 0.331476 1.907754 1.266479	C 3.436094 0.897437 3.315667
C -0.715336 -3.382584 -1.773818	C 1.125278 0.955157 2.285792
C 2.505288 2.877275 1.776940	C -1.984897 2.283158 1.736742
C 1.622982 1.786575 1.794651	C -1.116195 3.264765 2.548382
C 3.882607 2.749771 2.383039	C -5.349642 -0.116715 -0.615454
C -2.315138 2.001143 2.738849	C 1.995772 0.445801 3.262833
C -0.060399 -3.447655 -0.388348	C -0.219797 0.563175 2.254809
C -1.438281 0.741653 2.621359	C -1.764916 -2.995437 0.172958
C 2.650227 -1.549215 0.651781	C -1.020564 -4.061228 1.001007
C 1.231353 -4.278984 -0.506839	C -3.144514 1.765473 2.606382
C 5.092362 -1.080092 1.115247	C -2.934607 -2.481364 1.019702
C -2.327840 -0.506344 2.612194	C 1.496964 -0.472905 4.196031
C 1.370268 -1.937247 1.059762	C -0.694569 -0.350112 3.210078
C -1.028860 -4.108204 0.607396	C 0.154058 -0.881116 4.187917
C 3.710927 -1.483871 1.573107	C -0.376194 -1.855744 5.213656
C -0.498646 0.665845 3.841768	H 0.676142 0.895920 -5.204566
C 1.145959 -2.266528 2.410542	H 1.596220 1.695612 -3.909962
C 3.459677 -1.810911 2.908410	H -1.346457 2.393136 -4.725529
C 2.183206 -2.210620 3.344817	H 1.406363 -0.077261 -3.903302
C 1.948756 -2.566354 4.793623	H 1.665770 5.767082 -1.373972
H -0.530385 3.174350 -4.004624	H -0.420823 3.189142 -3.440972
H -1.172827 0.970752 -5.086884	H 2.134408 -2.819851 -4.321505
H -2.583099 2.162276 -3.393590	H -1.476732 -0.088952 -4.879327
H 1.059421 2.524862 -3.549596	H 5.089688 0.647286 -1.624632
H 0.405421 0.288309 -4.651017	H 3.455085 -3.826607 -3.693338
H 0.007492 5.424437 -0.974206	H 2.873772 5.644402 -0.082331
H -0.002498 3.230613 -2.302139	H 4.138326 3.635729 -0.218532
H -1.098684 -0.543919 -4.156320	H 5.478528 1.674527 -0.230547
H 1.136335 6.285969 0.090317	H -2.014762 2.469893 -3.076377
H -2.204528 2.056618 -1.665527	H 1.809436 -4.477321 -3.798279
H -0.518316 5.950400 0.630675	H -0.889041 -1.121983 -3.560110
H 4.597147 3.109363 -2.113927	H -0.032077 3.507972 -1.252801
H -2.709309 0.600106 -2.573863	H 1.147524 5.455564 0.287709
H 3.306890 -2.326438 -4.814169	H -2.322572 -0.092833 -3.323711
H 5.804073 1.858041 -1.762572	H 2.326034 -0.085473 -1.424376
H -5.654582 2.733816 0.641452	H 4.639562 0.121142 -0.001053
H -7.901524 1.668616 0.728436	H 4.115225 -3.396521 -1.446021
H 2.066200 2.146815 -1.143632	H 0.025854 -2.673001 -2.558774
H 5.261304 -0.171375 -2.870688	H -1.743063 3.396906 -0.123964
H 1.199179 -1.551325 -3.160270	H 4.906422 -2.922695 0.739028
H 4.846899 -2.256279 -3.934731	H -4.249801 -0.129583 -2.487781
H -1.086190 3.209549 0.292587	H -4.328191 1.457355 -1.708719
H 2.754839 4.935419 1.186111	H 4.032238 -1.681496 1.668400
H -3.676032 1.393108 -0.061023	H -3.235688 2.425165 -0.059805
H -8.176200 -0.721667 0.087174	H 3.928343 0.776562 2.340753
H 4.685100 2.413385 -0.493357	H 1.487601 1.673370 1.548592
H 3.477775 -3.164106 -3.266718	H -3.108681 3.918787 0.884394
H -6.184746 -2.052617 -0.631047	H -1.491106 -4.073048 -1.715918
H -0.044917 -2.913688 -2.504080	H -2.806255 -2.864098 -1.746114
H -1.676619 -2.859375 -1.758849	H -0.272040 3.631092 1.947964
H -2.998817 2.095278 1.886382	H 3.504902 1.963270 3.577337
H 4.479226 3.655356 2.220529	H 3.708558 -3.392101 1.961265
H -0.914318 -4.407376 -2.116325	H 1.320275 -1.878015 1.464705
H -1.708067 2.912129 2.816452	H -6.306544 0.154845 -1.080727
H 2.826450 -1.314333 -0.397994	H -1.735030 4.126755 2.836483
H 4.426944 1.895174 1.956943	H 4.005392 0.331539 4.063155
H 5.067982 -0.114337 0.590664	H -3.022336 -4.412453 -0.891153
H 1.942777 -3.808739 -1.199888	H -5.325888 -1.206785 -0.499186

H -3.117065 -0.442032 1.856470	H -0.203449 -4.532515 0.440050
H -2.925784 1.924811 3.650102	H -5.306515 0.346256 0.378331
H 1.935262 0.835066 2.226414	H -0.720847 2.807648 3.464402
H 3.820626 2.577923 3.467318	H -3.498961 -1.701144 0.502292
H 5.503802 -1.814271 0.407549	H -3.742810 1.025132 2.057097
H 0.967064 -5.267964 -0.907440	H -3.801676 2.602917 2.882610
H -1.947047 -3.515343 0.711591	H -0.606958 -3.625696 1.921455
H 0.123241 1.564008 3.939845	H -2.575633 -2.080787 1.975452
H 5.787659 -0.999382 1.959456	H -1.737434 -4.845378 1.284904
H -1.741274 -1.419312 2.442644	H -3.620166 -3.312017 1.236088
H -1.312875 -5.102222 0.233042	H -2.779982 1.308709 3.534362
H -2.816317 -0.601764 3.591188	H 2.169729 -0.878111 4.955953
H 1.729743 -4.423456 0.460531	H -1.740674 -0.651003 3.182836
H 0.155050 -0.213356 3.783843	H -0.835476 -2.732416 4.735527
H -0.566160 -4.245266 1.592414	H 0.419855 -2.211782 5.878123
H -1.116980 0.581981 4.747117	H -1.149472 -1.387255 5.839286
H 0.150255 -2.565666 2.733456	N -0.130099 0.884113 -1.850425
H 4.277429 -1.760619 3.632249	N -2.885227 0.183090 -0.964963
H 2.166898 -1.713345 5.452364	N -0.816942 -1.842097 -0.143324
H 0.912098 -2.875157 4.972170	N -1.116702 1.123286 1.262541
H 2.605313 -3.389900 5.106997	Mo -1.229514 0.077606 -0.414323
N 0.064279 0.520635 -1.878392	
N -2.357841 -0.773150 -0.549627	
N -0.594196 0.796431 1.344038	
N 0.299140 -2.033453 0.087066	
O -3.797189 -2.396972 -1.248894	
Mo -0.663218 -0.349250 -0.262580	
**$MoNCH_2$**	**TiNK**
−573.34058861	−890.37555928
C 3.040250 4.455229 0.973730	C -3.975642 -0.667910 5.491597
C 1.752035 3.801368 0.526259	C -5.466354 -2.707644 -0.115032
C 4.039658 -0.301919 1.744197	C -5.233306 -3.190148 -1.532878
C 0.520744 4.231049 1.047004	C -5.685991 -0.672001 1.110980
C 1.757936 2.750915 -0.396948	C -2.867323 -0.162057 4.594893
C 4.015357 -0.700445 -0.712999	C -2.000816 -1.035787 3.927442
C 0.002064 1.776096 4.356756	C 0.616351 -3.098191 4.873204
C 3.391203 -1.136057 0.619458	C -4.930461 0.621252 1.399170
C -0.684275 3.631816 0.659161	C -2.640062 1.212101 4.393921
C 2.339918 1.309875 -3.560635	C -0.823410 -3.280948 1.965117
C 0.935857 0.385997 2.456244	C 0.012225 -4.531373 1.671135
C 0.289441 0.446820 3.696456	C -1.814631 -2.998707 0.834573
C -1.997163 4.124298 1.224892	C -3.546425 -3.546653 -3.173219
C 0.563909 2.129038 -0.814655	C -5.793796 2.008131 -0.379423
C 3.681404 -2.633330 0.847883	C -0.962614 -0.602806 3.086506
C 0.357897 2.859676 -3.507505	C 3.008193 -6.885692 -0.240143
C -0.648138 2.581737 -0.271796	C 1.329172 -2.347010 3.740049
C 1.227805 -0.845378 1.840870	C -1.633695 1.677986 3.557119
C -0.069828 -0.753680 4.329320	C -0.753956 0.796466 2.848675
C 0.843128 1.424620 -3.216764	C 2.132545 -1.170095 4.306735
C 0.847184 -2.027341 2.499430	C -5.301987 2.995526 -1.417238
C 0.201868 -1.997285 3.743854	C -2.269735 -2.909281 -3.679239
C -0.186281 -3.284037 4.437242	C 2.990400 -5.374290 -0.257664
C 0.033917 0.469775 -4.110589	C 3.629895 -4.626715 0.737518
C 1.915125 -2.571301 -3.066651	C 2.314595 -4.671148 -1.262016
C -3.852278 0.714183 2.782965	C 3.605902 -3.224162 0.746794
C -2.536868 -0.457709 0.962993	C 2.262389 -3.270685 -1.290919
C -3.573161 0.408757 1.328984	C 4.421050 -2.471186 1.766255
C -2.271657 -0.753430 -0.387303	C 1.921583 2.495342 4.017767
C -4.357388 0.984723 0.316285	C 2.900461 -2.526721 -0.265378
C -3.066427 -0.148933 -1.375883	C -3.889813 3.124161 -3.320726

C -4.117231 0.716995 -1.038189	C -1.324388 2.846509 0.146332
C -0.470659 -4.028715 -0.611023	C -1.423441 -0.818797 -4.488506
C -4.968460 1.348482 -2.116917	C 0.404136 2.664000 1.988724
C -1.600938 -3.084575 -1.052600	C 1.586597 -2.560358 -2.430348
C -1.837008 -3.224508 -2.569603	C 1.236581 3.299996 2.944895
C -2.875239 -3.499026 -0.290790	C -3.325769 2.324938 -4.475589
H 3.012083 5.542241 0.810739	C -0.311436 3.468988 1.070257
H 3.216919 4.294737 2.048006	C -1.905960 0.505733 -5.039890
H 3.905235 4.055802 0.430176	C 1.381112 4.693113 2.923875
H 5.127951 -0.466232 1.739526	C -0.131328 4.861132 1.078180
H 0.503477 5.052217 1.770302	C 0.716414 5.498369 1.990433
H 3.852439 0.770703 1.590159	C 4.002739 -0.581712 -1.016538
H 2.706388 2.389336 -0.794335	C 5.096794 -1.387566 -1.464073
H 3.657231 -0.577829 2.736679	C 0.915937 6.996589 1.969860
H 5.109567 -0.796623 -0.658070	C 4.289407 2.748927 1.193059
H 0.522917 1.860339 5.322256	C 1.686921 1.460033 -3.490572
H 0.324032 2.609153 3.720404	C 4.068723 0.799084 -1.377675
H 3.770716 0.347355 -0.929339	C 1.070616 3.671947 -2.429697
H 2.937335 2.023176 -2.976313	C 3.415332 3.301080 0.057601
H 0.927836 3.616054 -2.949931	C 2.142947 2.582822 -2.551270
H 4.766889 -2.819322 0.821423	C 6.163716 -0.857852 -2.179491
H 1.234231 1.306087 1.951208	C 5.152347 1.297722 -2.119250
H -2.178667 5.174508 0.951070	C 6.228624 0.503675 -2.527114
H -1.072303 1.898057 4.558195	C 4.179230 4.368384 -0.740845
H -1.998762 4.073066 2.324031	C 7.406132 1.081645 -3.278788
H 2.506096 1.529116 -4.627133	H -3.832565 -1.728577 5.740870
H 3.656083 -1.319692 -1.540487	H -4.971732 -0.575886 5.025183
H 0.478315 3.064166 -4.581889	H -4.018042 -0.109095 6.438330
H 2.700089 0.294739 -3.353947	H -6.542680 -2.829313 0.110549
H 3.310263 -2.965885 1.826627	H -5.513285 -4.261077 -1.582195
H -0.568185 -0.720135 5.302633	H -4.889123 -3.318293 0.602146
H -0.706063 2.976681 -3.254313	H -5.633582 -1.295078 2.022993
H 3.206111 -3.237214 0.062572	H -6.755228 -0.488213 0.889164
H -2.836412 3.523605 0.855077	H -5.881631 -2.631954 -2.235381
H -1.575518 2.105233 -0.593790	H -2.139117 -2.107543 4.085377
H 2.872022 -2.306067 -3.529039	H 0.074546 -3.987494 4.522659
H 1.052774 -2.984170 2.019345	H -1.393300 -3.460804 2.890644
H 0.694342 -3.915585 4.624743	H -0.095248 -2.441886 5.395502
H -3.808370 1.796005 2.977620	H -0.660965 -5.386659 1.496109
H -0.668711 -3.086822 5.403136	H 1.357753 -3.437711 5.614632
H 0.175308 0.738795 -5.168406	H -2.402427 -3.902943 0.612822
H -3.121170 0.222061 3.435717	H -5.503906 1.204479 2.144976
H 0.348471 -0.570756 -3.975290	H -3.272163 1.943430 4.906465
H -1.036384 0.549863 -3.875310	H -3.417744 -4.643238 -3.104061
H -1.915853 -0.924232 1.730395	H 0.676812 -4.800591 2.502286
H -0.883498 -3.875689 3.826514	H 2.154666 -7.290556 0.327122
H 1.431018 -3.509434 -3.355577	H -6.427141 1.241046 -0.861503
H -4.856548 0.373603 3.076524	H -2.510229 -2.192380 1.105431
H -5.174182 1.659558 0.588671	H -6.404567 2.548850 0.369715
H 0.477650 -3.789052 -1.106114	H -3.947966 0.395609 1.836558
H -4.358635 1.943426 -2.812102	H -4.361967 -3.334550 -3.889032
H -0.324126 -3.958237 0.475341	H 3.923400 -7.271927 0.228607
H -2.845306 -0.355333 -2.423776	H 0.631000 -4.399325 0.773112
H -5.731654 2.009722 -1.686916	H -1.283729 -2.693264 -0.077906
H -0.945907 -2.903081 -3.125228	H 2.947697 -7.297692 -1.256789
H -0.734223 -5.068503 -0.855928	H 2.026523 -3.037410 3.235670
H -2.739199 -3.396328 0.795601	H 1.469516 -0.455429 4.814484
H -5.485527 0.583417 -2.714240	H -6.150948 3.606336 -1.781075
H -2.688012 -2.608383 -2.891049	H 2.872141 -1.528921 5.039600
H -3.748078 -2.899840 -0.585730	H -1.508602 2.754316 3.436178

H -2.059433 -4.270611 -2.833993	H 4.186931 -5.146629 1.522497
H -3.095553 -4.554705 -0.510092	H -1.492495 -2.930189 -2.894922
N 1.903618 -0.884554 0.568265	H -1.896223 -3.482908 -4.549830
N 0.583749 1.051919 -1.768917	H 1.825380 -5.228231 -2.067001
N 1.362122 -1.781959 -2.187429	H -4.562813 3.670062 -0.947826
N -1.212929 -1.660084 -0.728745	H 1.184749 2.077055 4.720393
Mo 0.579866 -0.766738 -0.928747	H 2.673512 -0.626976 3.521458
	H 4.610337 -3.082086 2.658948
	H -2.214806 2.527012 0.709564
	H 1.100302 -3.281628 -3.103134
	H 3.926293 -1.539897 2.060626
	H 0.851215 -1.832073 -2.042099
	H 2.462698 1.641056 3.595241
	H -4.486133 3.959549 -3.737926
	H -0.917266 1.946908 -0.342293
	H 2.626455 3.115100 4.586738
	H -4.151031 1.878741 -5.062343
	H -0.723100 -0.668980 -3.646205
	H -0.886419 -1.358014 -5.291677
	H -3.067577 3.556194 -2.720396
	H -1.650406 3.569783 -0.613430
	H -2.672672 0.316922 -5.813765
	H 5.395412 -2.181927 1.341660
	H 2.319023 -1.984704 -3.016581
	H -2.768073 3.017967 -5.135805
	H 2.030130 5.161921 3.669450
	H -1.058111 1.040040 -5.506679
	H 5.090868 -2.456224 -1.250184
	H -0.690819 5.465090 0.357129
	H 3.758697 1.991343 1.785018
	H 0.836710 0.920354 -3.046357
	H 0.113646 3.234878 -2.116105
	H 0.924877 7.414561 2.986401
	H 2.532106 3.782503 0.508269
	H 0.119607 7.498287 1.404379
	H 4.580395 3.562994 1.875506
	H 2.493469 0.737924 -3.672565
	H 1.335953 4.455322 -1.707640
	H 1.377749 1.884173 -4.460359
	H 5.203105 2.284899 0.794324
	H 0.915666 4.152338 -3.409856
	H 1.874427 7.268548 1.500633
	H 6.969276 -1.530286 -2.488474
	H 3.062972 3.031387 -2.961049
	H 4.417859 5.211113 -0.072145
	H 3.599846 4.775164 -1.580734
	H 5.150103 2.349395 -2.411304
	H 5.132899 3.985018 -1.129066
	H 7.812392 0.364289 -4.006369
	H 8.231246 1.354449 -2.599322
	H 7.125109 1.991736 -3.827494
	N 0.241208 1.243277 2.001143
	N -0.101181 -0.334625 -0.706537
	N 2.917383 -1.096009 -0.321331
	O -5.077273 -1.335813 -0.001556
	O -3.863198 -3.005329 -1.884534
	O -4.650139 1.418710 0.243740
	O -4.702540 2.280162 -2.503142
	O -2.574224 -1.564182 -4.062127
	O -2.467567 1.296144 -3.982319

	P 0.229095 -1.756387 2.310511<br>P 2.661548 1.848945 -0.890913<br>K -2.723888 -0.153509 -1.375865<br>Ti 1.126450 -0.040739 0.385119
**TiN**	**TiNH**
−658.84924335<br>C 2.325459 6.247640 -1.590456<br>C 1.080544 4.876342 0.148252<br>C 3.395246 2.746908 2.313174<br>C 1.395699 5.121676 -1.195281<br>C -0.137020 3.617364 1.980933<br>C 0.260852 3.807986 0.539872<br>C 3.606208 1.254780 2.025721<br>C 4.864149 1.920772 -0.930728<br>C 0.843288 4.271462 -2.161387<br>C 3.263004 0.394745 3.250203<br>C 3.348728 1.681317 -0.913235<br>C -0.247133 2.927521 -0.448233<br>C 0.026967 3.187171 -1.813200<br>C -2.989948 3.330049 -0.321683<br>C -4.361548 3.543787 -0.271451<br>C 2.887302 1.052866 -2.235986<br>C 4.699194 -1.297350 0.357378<br>C -2.405375 2.053386 -0.034120<br>C 6.728842 -2.825776 0.210495<br>C -0.598354 2.326341 -2.881310<br>C 3.328900 -1.014139 0.251780<br>C -5.264998 2.521781 0.079229<br>C 5.245784 -2.557199 0.088261<br>C -6.757760 2.758506 0.125906<br>C -3.170958 -0.369615 3.312349<br>C -3.328399 1.011932 0.303699<br>C -4.706005 1.271556 0.366624<br>C 2.399677 -2.042151 -0.109890<br>C 4.340032 -3.557731 -0.313473<br>C -3.539855 -1.245272 2.106636<br>C 2.974663 -3.319714 -0.410667<br>C 0.243105 -3.629883 1.917496<br>C -3.293418 -2.730671 2.401567<br>C 0.233170 -2.904504 -0.506116<br>C -2.968913 -1.047202 -2.174389<br>C 0.435372 -2.257428 -2.946816<br>C -3.355726 -1.694812 -0.836994<br>C -0.214480 -3.806401 0.492867<br>C -0.115822 -3.143099 -1.857877<br>C -4.860339 -1.994771 -0.800087<br>C -1.034914 -4.882826 0.125922<br>C -0.935599 -4.233188 -2.180556<br>C -1.417308 -5.112090 -1.202515<br>C -2.344275 -6.250526 -1.566891<br>H 2.247385 7.097426 -0.897956<br>H 3.378112 5.920201 -1.586769<br>H 2.104100 6.613074 -2.603161<br>H 1.476032 5.543696 0.920305<br>H 4.069038 3.065909 3.126348<br>H 0.414082 4.306103 2.636259<br>H 3.597291 3.381746 1.439204<br>H 2.364703 2.937792 2.637865<br>H 5.227941 2.412502 -0.016875<br>H 3.775575 0.795254 4.141894	−721.14152651<br>Ti -0.230512 0.045394 -0.637968<br>N 1.337684 -1.231826 -0.241694<br>N -2.132137 -0.441451 -0.090062<br>N -0.228319 0.398888 -2.303631<br>N 0.566912 1.789340 0.224958<br>H -0.600707 -5.451526 -1.915628<br>H 1.198957 -4.930424 -3.042179<br>H 3.657130 -4.933463 -3.353185<br>H -1.977413 -4.644247 -1.149689<br>H -0.696141 -3.690519 -3.797408<br>H -0.488449 -4.934453 -0.218291<br>H -2.075103 -2.918551 -2.973055<br>H -7.284750 -1.371450 -3.538979<br>H 5.012556 -3.124890 -2.333621<br>H -3.003794 -4.073838 2.630795<br>H 1.385653 -4.381883 2.367874<br>H 1.379180 -3.937875 0.652971<br>H -0.637259 -1.954906 -3.386733<br>H -2.898369 -2.967149 0.427617<br>H -0.713005 -2.539816 -0.946049<br>H 0.065902 -3.373344 1.725299<br>H -7.958977 -0.147041 -2.450508<br>H -7.080977 0.346604 -3.914491<br>H -4.517941 -0.183681 -4.030970<br>H -1.743064 -3.874784 5.139013<br>H 3.730591 -3.051256 0.923853<br>H 3.715372 -3.437234 2.656891<br>H -3.438348 -3.400205 5.312984<br>H 6.254031 -1.750614 -1.312329<br>H -6.745366 -0.526120 -0.365523<br>H -2.407517 -0.223099 -2.742824<br>H 5.653396 -2.125155 0.316499<br>H 1.874247 -2.969764 4.080611<br>H 4.857579 -0.492868 -2.960590<br>H -4.651488 -0.572929 0.906730<br>H -2.153272 -2.328835 5.908225<br>H 4.097937 -1.782167 2.128785<br>H -0.223454 0.679597 -3.286712<br>H 0.662049 -1.726738 3.705072<br>H 3.324922 0.264392 -2.480243<br>H 6.167252 -0.473861 -0.094159<br>H 3.736644 -0.461949 -0.103826<br>H 2.384853 -1.288255 3.942942<br>H 4.870733 0.893106 -1.841921<br>H -1.743975 -0.417121 4.521262<br>H 1.709093 2.335626 -3.541230<br>H 1.548301 0.039177 2.826374<br>H -1.649534 0.684777 2.311869<br>H 3.489954 2.332782 -3.506670<br>H -3.059406 2.439860 -1.017756<br>H 2.546110 1.971402 -1.225750<br>H -4.082908 2.546034 0.428801<br>H -1.731515 2.210787 1.093125<br>H 2.608872 3.866487 -3.647010

H	-1.213477	3.812729	2.105966
H	4.668154	1.102649	1.769993
H	5.124168	2.573705	-1.781210
H	0.019482	2.577222	2.311814
H	1.050296	4.456339	-3.220124
H	2.837039	2.652893	-0.800683
H	2.174251	0.384118	3.422517
H	5.415261	0.977585	-1.060876
H	-2.334283	4.162031	-0.583164
H	3.585033	-0.645442	3.106661
H	5.378368	-0.502357	0.675079
H	-4.743508	4.542836	-0.505823
H	7.247676	-1.972430	0.670227
H	3.130578	1.716277	-3.081778
H	-7.031331	3.555143	0.837245
H	-0.196974	2.571646	-3.873896
H	1.802176	0.885588	-2.243184
H	3.380030	0.082909	-2.401203
H	7.202489	-3.007020	-0.769558
H	-3.497138	0.668537	3.163528
H	-1.690536	2.463215	-2.901675
H	6.935676	-3.710814	0.833704
H	-2.078351	-0.358528	3.459911
H	-0.431588	1.259292	-2.683748
H	-7.157967	3.057234	-0.857686
H	-3.662906	-0.759753	4.220004
H	-7.291878	1.847538	0.431664
H	-5.381202	0.462609	0.656128
H	0.103018	-2.591825	2.262743
H	4.714109	-4.560301	-0.545547
H	-4.610960	-1.108640	1.880225
H	1.323090	-3.829951	1.997057
H	0.448027	-1.207114	-2.632012
H	-1.894266	-0.826360	-2.218317
H	-2.246315	-2.899170	2.684344
H	-3.512855	-0.102266	-2.322761
H	-0.283532	-4.322354	2.588622
H	2.314981	-4.136453	-0.708368
H	-3.926067	-3.051216	3.246245
H	1.481414	-2.520391	-3.170744
H	-5.455051	-1.079027	-0.930808
H	-3.520392	-3.379538	1.544342
H	-0.148404	-2.352830	-3.872335
H	-2.806174	-2.647496	-0.747417
H	-3.209085	-1.723310	-3.011280
H	-5.174434	-2.483446	0.133195
H	-5.119062	-2.674328	-1.630054
H	-1.379206	-5.568222	0.906549
H	-1.203645	-4.398650	-3.228864
H	-3.392050	-5.913649	-1.626731
H	-2.303538	-7.055221	-0.819985
H	-2.087106	-6.681988	-2.544896
N	-1.050114	1.816020	-0.077297
N	0.038925	-0.020358	2.331522
N	1.046306	-1.792072	-0.157082
P	2.639580	0.658342	0.517591
P	-2.623107	-0.652554	0.567568
Ti	0.010571	0.000686	0.679196
H	3.247956	1.153901	3.239350
H	4.739222	3.162735	-1.497451
H	0.815402	1.265576	4.160794
H	-3.650539	4.024375	-0.463680
H	3.769909	2.311476	1.998819
H	-0.309463	2.486379	3.525677
H	3.891803	3.720192	-0.037771
H	-3.184944	3.544161	2.576891
H	3.896782	4.725694	-1.500486
H	3.442446	2.871530	3.654902
H	1.814865	5.451565	-2.322579
H	1.060692	2.982832	4.539645
H	2.202530	4.262639	1.300545
H	-1.532049	4.198313	2.675706
H	-1.984784	5.620420	-0.355967
H	-2.766701	5.038802	1.720052
H	0.575165	4.368990	1.989873
H	-0.247093	6.706600	-1.753884
H	1.993318	4.543104	3.039641
C	-0.879039	-4.658642	-1.205732
C	1.804384	-4.168692	-2.548763
C	3.181747	-4.171690	-2.734498
C	-0.978311	-2.939463	-3.043863
C	-0.359520	-3.285462	-1.674207
C	1.163194	-3.212467	-1.746288
C	3.938552	-3.161408	-2.148692
C	-7.098761	-0.381738	-3.091504
C	1.144449	-3.575064	1.658902
C	-2.697639	-3.026664	2.571216
C	1.951764	-2.226614	-1.088547
C	3.353461	-2.169998	-1.348589
C	-2.629635	-2.411668	1.325982
C	-5.807948	-0.366837	-2.305088
C	-4.558857	-0.274559	-2.942642
C	-2.430266	-3.016204	5.098836
C	3.472225	-2.660943	1.914097
C	-5.797713	-0.471874	-0.907580
C	-3.362949	-0.305563	-2.228826
C	1.968725	-2.324037	2.025784
C	-2.371078	-2.332153	3.753329
C	-4.605937	-0.503072	-0.180560
C	5.651443	-1.367208	-0.476139
C	-3.350515	-0.432296	-0.825694
C	4.229594	-0.989626	-0.931405
C	-2.226564	-1.062683	1.194939
C	1.699258	-2.038662	3.522500
C	1.564011	-1.112367	1.102570
C	4.322509	-0.018131	-2.123791
C	-1.984882	-0.994404	3.625824
C	-1.916774	-0.368942	2.373599
C	1.389850	0.123260	1.761458
C	2.593049	2.873546	-3.174269
C	1.167814	1.493633	1.382367
C	-3.276950	3.044994	-0.128235
C	2.566176	2.988014	-1.636706
C	0.339208	3.119632	-0.313801
C	-2.029843	3.211829	0.760164
C	1.298777	3.701168	-1.179548
C	3.843588	3.689059	-1.135099
C	3.110848	2.176685	2.868352

	C 1.638175 2.495477 2.501196<br>C -0.876750 3.801966 -0.045033<br>C 0.745246 2.281405 3.752375<br>C 1.067694 4.993950 -1.671989<br>C -2.389405 4.045593 2.005952<br>C -1.056528 5.089636 -0.572299<br>C 1.589708 3.999959 2.170782<br>C -0.089720 5.700787 -1.362844
**ZrNH**	**ThNTh**
−662.35637554<br>C 2.165487 -6.227521 -1.604930<br>C 1.285396 -5.061343 -1.217648<br>C 0.789267 -4.175570 -2.181691<br>C 4.875502 -2.078336 -1.044617<br>C 0.914354 -4.849274 0.116399<br>C 2.940331 -1.075189 -2.323327<br>C -0.597641 -2.208020 -2.925100<br>C -0.033441 -3.092777 -1.843083<br>C 3.375443 -1.757901 -1.018613<br>C 0.086574 -3.786246 0.502117<br>C -0.369491 -2.873115 -0.483848<br>C 3.373802 -2.854444 2.224929<br>C -3.152331 -3.181381 -0.539287<br>C 6.811543 2.765351 0.137501<br>C 3.734149 -1.393401 1.920492<br>C -4.532216 -3.349276 -0.584161<br>C 0.343677 2.004297 -2.982040<br>C 4.796846 1.221912 0.270416<br>C -0.363750 -3.656975 1.936017<br>C 5.332447 2.486450 -0.004111<br>C 3.424765 0.941595 0.181519<br>C -2.560105 -1.947497 -0.150902<br>C 4.422118 3.477899 -0.402789<br>C -5.421228 -2.315873 -0.235245<br>C 2.495179 1.967729 -0.168049<br>C -6.919906 -2.512127 -0.270849<br>C 3.549328 -0.500446 3.153578<br>C 3.053314 3.235737 -0.484143<br>C -0.099289 2.990994 -1.931526<br>C -3.336783 1.386684 -2.010964<br>C -3.457038 -0.897521 0.198749<br>C -4.845905 -1.104018 0.159989<br>C -0.874229 4.099048 -2.292598<br>C 0.302298 2.830358 -0.582259<br>C -3.427537 1.960632 -0.589132<br>C -1.270549 5.065096 -1.357346<br>C -0.068013 3.807578 0.374419<br>C -4.839243 2.493217 -0.305165<br>C -2.108275 6.251753 -1.774321<br>C -0.856785 4.895221 -0.031938<br>C 0.436811 3.733868 1.792748<br>C -3.332865 0.012902 3.311390<br>C -3.513747 1.144945 2.291820<br>C -2.967570 2.476120 2.827249<br>H 2.634695 -6.067637 -2.584225<br>H 1.584419 -7.160650 -1.666628<br>H 2.963349 -6.391925 -0.867697<br>H 1.049693 -4.328848 -3.232404<br>H 5.098476 -2.696537 -1.928955<br>H 3.127661 -1.743767 -3.178002	−867.69759848<br>C -3.322438 -4.276542 1.498796<br>H -2.725144 -4.545779 2.383325<br>H -2.646526 -4.293807 0.631766<br>H -4.078248 -5.064877 1.349312<br>C -1.784534 -2.554964 -4.421539<br>H -1.049986 -1.765193 -4.629192<br>H -2.640161 -2.404245 -5.096928<br>H -1.323924 -3.520639 -4.684685<br>C -3.557910 -4.009441 -2.459454<br>H -3.899299 -4.166800 -1.426509<br>H -3.041655 -4.929957 -2.780727<br>H -4.446893 -3.888566 -3.094265<br>C -0.877493 -3.061400 -1.506701<br>H 0.001016 -2.411900 -1.609887<br>H -0.572506 -4.089696 -1.756865<br>H -1.143671 -3.075612 -0.436976<br>C -5.324970 -1.409675 -4.004969<br>H -4.819355 -2.087116 -4.707743<br>H -6.044141 -0.808345 -4.586381<br>H -5.901518 -2.023056 -3.296450<br>C -3.330965 0.836899 -4.452571<br>H -2.656337 1.598932 -4.036318<br>H -4.089204 1.357510 -5.060140<br>H -2.733115 0.206024 -5.127943<br>C -5.306023 0.879937 -2.079191<br>H -4.775126 1.585023 -1.424206<br>H -5.959676 0.255937 -1.450373<br>H -5.951720 1.467900 -2.751309<br>C -0.878000 2.841222 -1.901083<br>H -0.000060 2.601512 -1.287784<br>H -0.571399 3.574309 -2.663678<br>H -1.146497 1.924593 -2.451599<br>C -3.559334 4.140088 -2.240431<br>H -3.901295 3.324471 -2.893289<br>H -3.044783 4.879503 -2.877279<br>H -4.447957 4.628362 -1.816393<br>C -1.782981 5.110242 -0.000742<br>H -1.320887 5.821163 -0.704293<br>H -1.049690 4.893941 0.787921<br>H -2.638853 5.619932 0.466814<br>C -3.329505 3.435215 2.952422<br>H -4.087879 3.699780 3.707350<br>H -2.731229 4.335769 2.745786<br>H -2.655388 2.692582 3.402939<br>C 0.875005 -2.942293 1.731151<br>H -0.002405 -2.285314 1.783921<br>H 0.568113 -3.947880 2.059184<br>H 1.141481 -3.038874 0.665767<br>C 5.304438 1.225842 -1.898144<br>H 4.773138 0.286796 -2.108105

H	-0.102746	-2.393285	-3.886502	H	5.961085	1.059629	-1.030225
H	5.206086	-2.642406	-0.161567	H	5.947135	1.448152	-2.765227
H	5.481708	-1.164148	-1.124658	C	3.328240	3.214863	-3.195963
H	1.269046	-5.540315	0.885968	H	2.653123	2.442313	-3.591525
H	2.811312	-2.700965	-0.918090	H	4.085644	3.424874	-3.968920
H	3.501911	-0.143309	-2.485082	H	2.730586	4.128092	-3.052964
H	-1.676201	-2.387736	-3.051406	C	5.324082	4.106083	-1.088520
H	1.870108	-0.828597	-2.321654	H	5.901247	3.865868	-0.183116
H	3.489499	-3.510488	1.351792	H	4.818869	5.068432	-0.923995
H	-0.494267	-1.143935	-2.676064	H	6.042779	4.248135	-1.913221
H	-2.505211	-4.016089	-0.811154	C	0.881940	2.966453	1.689390
H	7.421713	2.010786	-0.380901	H	0.002641	2.686557	1.095359
H	-4.931577	-4.317129	-0.899095	H	0.577778	3.751246	2.399685
H	4.791169	-1.351028	1.611314	H	1.149221	2.089793	2.302160
H	4.025135	-3.238871	3.025314	C	1.783712	5.095451	-0.366589
H	5.479192	0.423785	0.571614	H	1.048413	4.824306	-1.136147
H	1.438595	1.919436	-3.008004	H	2.638143	5.571408	-0.870939
H	-0.009679	2.305262	-3.976230	H	1.323240	5.853617	0.286803
H	-0.041931	0.994566	-2.780584	C	5.301434	-2.261129	-0.114330
H	0.107312	-4.425555	2.562356	H	5.956431	-1.425310	-0.405046
H	2.333983	-2.934341	2.570946	H	5.945872	-3.122567	0.124381
H	7.070923	3.747541	-0.279308	H	4.772008	-1.974724	0.805326
H	7.124809	2.764252	1.193784	C	3.319337	-4.376286	-1.181723
H	-1.455272	-3.778526	2.006202	H	2.642108	-4.328647	-0.316864
H	-7.236192	-3.030315	-1.187875	H	4.074592	-5.151542	-0.972643
H	-0.139129	-2.664561	2.356403	H	2.723225	-4.710703	-2.044523
H	-2.343221	0.969922	-2.226165	C	5.318058	-3.005838	-3.011587
H	4.792378	4.476275	-0.650329	H	4.811805	-3.343078	-3.927260
H	-4.074577	0.585368	-2.160740	H	6.033635	-3.793687	-2.721415
H	4.121208	-0.914012	3.999294	H	5.898917	-2.104011	-3.256408
H	-3.530998	2.182070	-2.747028	C	0.880362	-0.021213	-3.414458
H	-7.274171	-3.115752	0.580037	H	0.001309	-0.394242	-2.873713
H	3.898902	0.523565	2.969279	H	0.575367	0.200854	-4.449153
H	-1.168262	4.214035	-3.339463	H	1.149150	0.947875	-2.962720
H	2.390377	4.045989	-0.789056	C	3.561447	-0.472223	-4.680049
H	-7.449020	-1.550233	-0.233046	H	3.901474	0.526950	-4.372883
H	2.489117	-0.449700	3.436438	H	3.048507	-0.362951	-5.650605
H	-5.508355	-0.285839	0.450681	H	4.451156	-1.096379	-4.842979
H	-2.721295	2.804417	-0.513010	C	1.781560	-2.867560	-4.228766
H	0.153002	0.022536	3.577726	H	2.636472	-3.541425	-4.389808
H	-5.600147	1.702878	-0.374606	H	1.319318	-2.681783	-5.211412
H	-1.623295	6.817884	-2.583127	H	1.047741	-3.399110	-3.607953
H	-3.821498	-0.912243	2.978872	N	-0.000051	0.001036	-0.000730
H	-5.093842	3.252862	-1.061295	N	-2.948685	-1.300498	1.902768
H	-3.094578	5.937986	-2.147991	N	-2.950256	-0.997342	-2.075064
H	-2.265655	-0.199788	3.465791	N	-2.947538	2.299655	0.172992
H	1.508595	3.984585	1.829956	N	2.949957	2.278803	-0.342154
H	0.342664	2.721749	2.212278	N	2.947306	-1.437417	-1.802839
H	-4.589456	1.256498	2.077814	Si	-2.337420	-2.553792	-2.602636
H	-4.924086	2.973625	0.678867	Si	-4.144206	-0.218923	-3.097308
H	-2.271941	6.939391	-0.934702	Si	-2.336459	3.535330	-0.910530
H	-1.137300	5.643879	0.713621	Si	-4.142467	2.793212	1.358809
H	-1.902856	2.376543	3.081349	Si	4.143588	2.687021	-1.561408
H	-3.775185	0.309449	4.275809	Si	2.339650	3.588451	0.650816
H	-0.100090	4.442121	2.436494	Si	4.138035	-2.700083	-1.545099
H	-3.067192	3.297533	2.104057	Si	2.337466	-1.232550	-3.433714
H	-3.509079	2.763631	3.742117	Th	-2.132445	0.001222	0.000877
N	-1.180269	-1.753553	-0.110308	Th	2.132259	0.000194	-0.000826
N	1.121130	1.713893	-0.208011	C	3.554754	-3.818789	2.750069
N	0.112096	-0.020826	2.557887	H	4.446218	-3.650561	3.370402

P 2.764489 -0.738149 0.447561<br>P -2.720583 0.709658 0.641709<br>Zr 0.036696 -0.040948 0.701164	H 3.039011 -4.711425 3.142882<br>H 3.892003 -4.055194 1.730879<br>C -5.323363 4.175657 0.785421<br>H -6.042250 4.375457 1.597869<br>H -4.818348 5.124017 0.553215<br>H -5.900155 3.871412 -0.100783<br>C -5.303593 -2.244072 0.278591<br>H -5.959808 -1.390351 0.508090<br>H -4.773876 -2.023865 -0.658972<br>H -5.946775 -3.121319 0.102112<br>C -5.304362 1.360352 1.796308<br>H -5.958434 1.132034 0.940679<br>H -4.774179 0.438825 2.075297<br>H -5.949677 1.644723 2.643054<br>Si -2.339383 -0.976307 3.514455<br>C -0.882200 0.230242 3.406496<br>H -0.002755 -0.182332 2.895854<br>H -1.149814 1.163418 2.883995<br>H -0.578003 0.528254 4.422155<br>C -1.782591 -2.547915 4.427928<br>H -1.046086 -3.121462 3.849126<br>H -1.323203 -2.289927 5.395551<br>H -2.636396 -3.210360 4.635689<br>C -3.564140 -0.126970 4.701116<br>H -4.451309 -0.740080 4.913211<br>H -3.049986 0.057874 5.659505<br>H -3.908363 0.844599 4.319208<br>Si 2.335836 -2.355251 2.785361<br>C 1.784999 -2.219612 4.599772<br>H 1.321395 -3.161275 4.934607<br>H 2.642266 -2.022017 5.260832<br>H 1.053561 -1.413871 4.749003<br>Si -4.140310 -2.577596 1.737996<br>C -5.320222 -2.774355 3.222630<br>H -4.814276 -3.045970 4.160052<br>H -5.898905 -1.855923 3.402084<br>H -6.037652 -3.579436 2.990114<br>C 3.563246 4.287369 1.933360<br>H 4.449911 4.747111 1.474670<br>H 3.047652 5.068262 2.517661<br>H 3.908415 3.520443 2.641168<br>N 2.949348 -0.843062 2.143208<br>C 3.336998 1.161830 4.377403<br>H 2.661253 1.890802 3.907412<br>H 2.741135 0.583284 5.099759<br>H 4.096994 1.726196 4.942177<br>C 5.328093 -1.113787 4.093729<br>H 5.902188 -1.778777 3.431289<br>H 6.049416 -0.471447 4.626611<br>H 4.823911 -1.736409 4.846450<br>C 5.307564 1.026022 2.003502<br>H 5.955315 1.661483 2.628748<br>H 5.959311 0.356101 1.421737<br>H 4.776416 1.681176 1.298763<br>Si 4.146379 0.007506 3.102653
**$ThNH_2$**	**VN(O)**
−444.71195228<br>Si -3.331760 -1.305357 -0.987898<br>Si 0.445304 3.390818 -1.007278<br>C 0.699660 5.119522 -0.266105	−614.51868548<br>C -0.092981 5.599882 -1.185180<br>C 1.181343 5.106242 -0.917002<br>C -1.202625 4.823898 -0.868141

N	1.519984	-1.794238	-0.027286		C	3.630726	4.341335	0.743051
C	0.678425	-4.723473	0.150093		C	1.370860	3.849615	-0.324242
Th	-0.053536	-0.062308	-0.326355		C	-3.350032	3.507707	0.868433
N	-0.236784	0.008533	-2.581521		C	-1.073298	3.556742	-0.278291
C	-2.267917	-2.732490	-1.649122		C	2.797921	3.359133	-0.103396
Si	-3.142587	0.561866	1.411701		C	3.484347	3.092824	-1.456588
C	-4.010389	-0.306442	-2.452650		C	0.228580	3.075840	0.001472
C	-3.818949	-0.565254	2.780768		C	-2.344404	2.756018	-0.023330
C	-1.866660	1.665173	2.289032		C	-3.003094	2.366581	-1.360020
C	-4.548601	1.682727	0.809194		C	0.295542	2.838505	2.832967
N	-2.360400	-0.302799	0.092064		C	0.599444	1.659746	1.938038
Si	1.998708	2.279010	1.408324		C	-4.216143	0.194062	3.047533
C	-0.415825	-2.578798	2.067767		C	1.105994	0.496581	2.550277
C	1.537141	3.626396	2.662033		C	-2.234866	-0.723223	-3.869111
C	2.034649	0.681773	2.447876		C	4.846344	-0.370447	-2.121765
C	3.757185	2.635189	0.794818		C	5.014331	-0.159459	0.387822
N	0.878335	2.060614	0.073083		C	4.093379	-0.533566	-0.791727
C	2.448298	-3.535113	2.330572		C	-5.069015	-0.961622	0.447711
Si	3.076951	-1.727482	-0.843611		C	-3.211657	-0.551385	2.159071
Si	1.107178	-3.115496	1.055911		C	-5.497238	-1.290099	-0.842302
C	3.089961	-0.270817	-2.070219		C	-3.709258	-0.834815	0.745819
C	4.537813	-1.430615	0.331789		C	-4.554478	-1.531208	-1.839637
C	-4.834723	-2.126517	-0.169180		C	-2.763783	-1.020202	-0.305103
C	-1.405885	3.285667	-1.428589		C	-3.175046	-1.416531	-1.602163
C	1.441340	3.356227	-2.617184		C	1.687964	-0.675955	1.995778
C	3.452326	-3.292598	-1.842768		C	-2.182484	-1.740656	-2.714466
H	-2.825796	-3.279092	-2.426516		C	3.498810	-1.925302	-0.608856
H	-1.321506	-2.405525	-2.111091		C	2.410428	-1.574291	2.975749
H	-2.036755	-3.451912	-0.849465		C	-2.763312	-1.858156	2.845935
H	-5.343084	-2.754800	-0.919079		C	2.313739	-2.133609	0.142522
H	-5.573545	-1.401356	0.201935		C	-2.393709	-3.168101	-3.257737
H	-4.546694	-2.777343	0.668966		C	4.130076	-3.040922	-1.173887
H	-3.223290	0.201506	-3.031331		C	1.767904	-3.432067	0.300659
H	-4.704956	0.473334	-2.104502		C	0.471120	-3.721795	1.053250
H	-4.562214	-0.959485	-3.147802		C	-0.620161	-4.228464	0.092776
H	-2.026172	3.526404	-0.552137		C	3.611049	-4.326300	-1.024016
H	-1.718442	2.299342	-1.805161		C	2.440106	-4.510604	-0.297757
H	-1.649826	4.016441	-2.216458		C	0.670488	-4.732392	2.200711
H	1.161251	4.205232	-3.261427		H	-0.220433	6.579094	-1.647890
H	1.256441	2.430737	-3.182025		H	2.054139	5.705690	-1.181149
H	2.522828	3.427250	-2.423077		H	-2.201642	5.201132	-1.091599
H	0.108180	5.280988	0.646260		H	3.775491	5.298125	0.219034
H	0.373353	5.863950	-1.011398		H	-3.686107	4.441475	0.393946
H	1.753310	5.334199	-0.032916		H	3.151301	4.560795	1.707982
H	2.200360	-0.244456	1.876237		H	3.541223	4.014154	-2.055657
H	1.111998	0.567393	3.040955		H	4.627611	3.922420	0.945753
H	2.859429	0.755755	3.175097		H	-2.924541	3.766492	1.848862
H	2.176139	3.511141	3.553567		H	-3.334594	3.261964	-1.908048
H	0.491297	3.530807	2.990806		H	0.908117	3.706519	2.552242
H	1.682640	4.645785	2.280109		H	-4.240439	2.884498	1.034057
H	4.463818	2.722594	1.635397		H	4.511427	2.730332	-1.301356
H	3.796455	3.580222	0.231766		H	-0.753166	3.146615	2.715576
H	4.123289	1.840881	0.127914		H	2.930512	2.344001	-2.038656
H	-0.733225	-3.416030	2.709942		H	2.751566	2.405089	0.438312
H	-1.297786	-2.295188	1.470413		H	0.483556	2.595290	3.884003
H	-0.180434	-1.740126	2.744718		H	-2.296312	1.823691	-2.001042
H	3.339365	-3.987731	1.871107		H	-3.877088	1.722325	-1.187977
H	2.047147	-4.265948	3.051985		H	-2.072314	1.826604	0.495460
H	2.771723	-2.649658	2.897552		H	-4.553964	1.131125	2.584434
H	1.567296	-5.161584	-0.328136		H	5.132691	0.680555	-2.262531

H -0.072847 -4.563902 -0.637145<br>H 0.279525 -5.470577 0.855187<br>H 5.467728 -1.339519 -0.252780<br>H 4.677301 -2.250793 1.050385<br>H 4.419323 -0.501443 0.908463<br>H 4.367131 -3.150690 -2.441231<br>H 2.630211 -3.533066 -2.533246<br>H 3.617022 -4.168327 -1.196937<br>H 2.656025 0.667328 -1.683014<br>H 2.584462 -0.523599 -3.014603<br>H 4.136748 -0.036617 -2.322305<br>H -3.040693 -1.249579 3.152579<br>H -4.674948 -1.173257 2.456994<br>H -4.152223 0.051914 3.631630<br>H -4.195669 2.385256 0.039564<br>H -4.957777 2.273083 1.644729<br>H -5.380891 1.106932 0.377455<br>H -1.351807 2.377945 1.630173<br>H -1.096566 1.078679 2.817338<br>H -2.388727 2.248340 3.064852<br>H -1.106041 0.063688 -3.116064<br>H 0.530156 -0.022961 -3.255521	H -1.980558 0.285987 -3.523903<br>H 5.407987 0.860190 0.260084<br>H -3.750123 0.441044 4.013072<br>H 3.249888 0.170984 -0.803555<br>H -3.234747 -0.694935 -4.329748<br>H 1.165124 0.547877 3.636596<br>H -2.315552 0.079090 2.060867<br>H -1.507661 -0.999012 -4.647295<br>H -5.103823 -0.418557 3.264556<br>H 4.223328 -0.669293 -2.975203<br>H -5.809011 -0.799974 1.232411<br>H -6.562076 -1.370108 -1.062262<br>H 4.485343 -0.196673 1.350018<br>H 5.773761 -0.962353 -2.144339<br>H 5.870721 -0.849331 0.445878<br>H -4.896044 -1.814300 -2.837348<br>H -1.172800 -1.682394 -2.289258<br>H 2.893928 -0.978782 3.758710<br>H -2.313734 -1.647971 3.829387<br>H -3.363531 -3.263533 -3.769524<br>H -3.623296 -2.528470 2.999422<br>H 3.158148 -2.198292 2.474390<br>H -2.022946 -2.388312 2.233706<br>H 1.689559 -2.245939 3.464431<br>H 5.040266 -2.899722 -1.756196<br>H -1.607940 -3.415044 -3.987089<br>H 0.107348 -2.781039 1.487379<br>H -2.364944 -3.917966 -2.455455<br>H -0.804459 -3.506999 -0.712178<br>H -1.566173 -4.385024 0.631398<br>H 1.467870 -4.427788 2.892531<br>H 4.113049 -5.178049 -1.484486<br>H -0.327989 -5.185769 -0.364528<br>H -0.261092 -4.841078 2.775360<br>H 2.025206 -5.514401 -0.195099<br>H 0.934881 -5.726659 1.811171<br>N 0.414935 1.766456 0.601236<br>N 0.526118 0.168853 -1.793534<br>N 1.627788 -0.978834 0.690001<br>O -1.460654 -0.804528 0.018905<br>V 0.166127 0.004967 -0.271824
**VN(N)**	**$MeNO_2$**
−654.01917050<br>C 0.144445 -0.554956 5.851805<br>C 0.029683 0.043478 4.469915<br>C 2.555963 -2.791124 2.485078<br>C 1.155873 0.555297 3.810812<br>C 4.632110 -1.296205 0.974871<br>C -1.200182 0.130062 3.814124<br>C 5.461281 -0.284515 0.492849<br>C -1.634624 -3.460069 2.520216<br>C 2.498524 -2.667404 0.953221<br>C 2.846428 -4.026433 0.310774<br>C 1.087257 1.085715 2.517731<br>C 2.302227 1.746583 1.922215<br>C 3.371991 -1.537566 0.410373<br>C 5.038811 0.503516 -0.574905<br>C -1.324540 0.662510 2.521099<br>C -0.157623 1.075686 1.835948<br>C -1.557832 -3.204201 1.006840	−47.17259485<br>1.O -3.195418 -1.628874 3.977583<br>2.C -2.181713 -0.411481 5.708057<br>3.N -2.173078 -1.493473 4.661762<br>4.O -1.131280 -2.151371 4.547735<br>5.H -1.607970 -0.769667 6.566497<br>6.H -3.220181 -0.180279 5.953678<br>7.H -1.684936 0.457088 5.260556

C -1.372632 -4.545986 0.268757<br>C 2.956851 -0.724762 -0.676440<br>C 3.791033 0.303917 -1.185665<br>C -2.699797 0.900258 1.957530<br>C -0.629183 3.796829 1.517457<br>C -0.857099 5.170564 1.404873<br>C -4.007362 -2.525444 1.052287<br>C -2.741152 -2.404321 0.464397<br>C -0.489240 2.983176 0.372508<br>C -5.102676 -1.808794 0.569956<br>C 3.027174 2.621280 -1.877054<br>C 3.382095 1.199972 -2.353580<br>C 2.802299 -2.229710 -3.043246<br>C 1.554221 -1.733054 -2.341456<br>C -0.959439 5.801202 0.159837<br>C -2.599582 -1.535825 -0.649897<br>C 4.468478 1.270512 -3.445608<br>C -1.214310 7.285754 0.039519<br>C -0.594781 3.616777 -0.882917<br>C 0.322772 -2.163680 -2.874905<br>C -0.823015 4.985991 -0.978278<br>C -0.977338 -2.112783 -2.328812<br>C -4.944610 -0.959378 -0.521613<br>C -3.703601 -0.807869 -1.160150<br>C -2.026993 -2.955376 -3.025781<br>C -3.738937 1.603601 -1.956322<br>C -3.598616 0.125564 -2.365388<br>C -4.638657 -0.213964 -3.453165<br>H 0.669809 0.123448 6.539188<br>H 0.713411 -1.496953 5.830422<br>H -0.843759 -0.771410 6.276722<br>H 3.540553 -3.140666 2.830228<br>H 2.120272 0.566046 4.325319<br>H 4.969316 -1.909275 1.810971<br>H 1.815306 -3.528978 2.825629<br>H 2.333581 -1.833814 2.974431<br>H 6.436119 -0.110346 0.951165<br>H 3.133252 1.735292 2.638122<br>H -0.695533 -3.914839 2.865882<br>H -2.105529 -0.199592 4.330082<br>H 3.901040 -4.285168 0.492887<br>H 2.222518 -4.821952 0.746049<br>H -1.785568 -2.528535 3.080858<br>H -2.443889 -4.160525 2.775553<br>H 2.083267 2.792147 1.661754<br>H 1.461513 -2.430811 0.676115<br>H 2.643790 1.253382 1.003996<br>H -0.518432 -5.095110 0.693253<br>H 2.675868 -4.027322 -0.773408<br>H 5.691653 1.294637 -0.946364<br>H -0.554571 3.352655 2.510247<br>H -0.657092 -2.601523 0.817265<br>H -4.138117 -3.185000 1.909939<br>H -2.268888 -5.176485 0.376988<br>H -0.954602 5.762006 2.317873<br>H -3.424699 0.183317 2.362201<br>H -3.037939 1.913531 2.226431<br>H 3.489528 -2.719954 -2.340447<br>H -1.177499 -4.406372 -0.801724<br>H 3.889724 3.104649 -1.393115	

H -2.722014 0.839867 0.865300<br>H -1.156819 7.779837 1.018343<br>H 5.367636 1.793211 -3.087116<br>H -6.077633 -1.910917 1.049332<br>H 2.192114 2.611369 -1.165424<br>H 3.353613 -1.389165 -3.488032<br>H 4.781302 0.274412 -3.790458<br>H 2.544690 -2.937968 -3.837562<br>H 2.469806 0.782382 -2.801260<br>H -0.481742 7.768071 -0.624195<br>H 2.723622 3.239670 -2.734337<br>H -2.212103 7.490782 -0.378334<br>H -2.653434 -3.499552 -2.307752<br>H 4.089936 1.828638 -4.314265<br>H -0.489816 3.009117 -1.780158<br>H 0.406698 -2.754186 -3.784751<br>H -2.952140 1.906407 -1.254042<br>H -5.804049 -0.399317 -0.893519<br>H -0.895007 5.435594 -1.972082<br>H -1.558657 -3.672265 -3.708604<br>H -4.715867 1.795721 -1.487068<br>H -2.698237 -2.310404 -3.611286<br>H -2.595130 0.013191 -2.798015<br>H -3.654552 2.247413 -2.844028<br>H -4.611574 -1.274345 -3.741739<br>H -5.661426 0.008550 -3.114020<br>H -4.451919 0.390340 -4.352894<br>N -0.245964 1.581108 0.481980<br>N 1.652395 -0.945484 -1.256727<br>N -1.293962 -1.381844 -1.247815<br>N -0.148151 0.956189 -2.198704<br>V -0.021134 0.181667 -0.833328	
**$SiMe_4$**	
–97.13151877<br>1.C 1.474073 -0.536123 -1.065162<br>2.C -1.604337 -0.577344 -0.821583<br>3.C -0.005627 1.892291 0.163442<br>4.C 0.140431 -0.772564 1.724881<br>5.H 1.435832 -0.067186 -2.061211<br>6.H -1.722433 -0.140239 -1.825609<br>7.H 1.478245 -1.627996 -1.212126<br>8.H -1.615001 -1.673366 -0.933065<br>9.H -0.125026 2.380228 -0.816821<br>10.H 2.434075 -0.258105 -0.602412<br>11.H -2.487975 -0.294747 -0.228193<br>12.H 0.935423 2.257183 0.604867<br>13.H -0.830248 2.234348 0.808751<br>14.H 0.175427 -1.871933 1.668829<br>15.H 1.050233 -0.435812 2.246373<br>16.H -0.723079 -0.498825 2.351926<br>17.Si 0.002131 0.003516 0.000835	

Supplementary Table 2. NMR Properties of **1*** computed with various functionals and scalar and spin-orbit relativistic effect in THF (before Empirical Scaling)

**1*** $^{15}N_{nitride}$	δ_{iso}	σ^{iso}	σ^{d}	σ^{p}	σ^{so}
BP86 SR	788.91	−921.44	342.56	−1264.00	NA
BP86 SOR	805.19	−937.35	342.56	−1239.92	−39.98
PBE0 SR	1121.88	−1275.11	340.14	−1615.25	NA
PBE0 SOR	1121.12	−1274.37	340.23	−1588.21	−26.39
PBE0 TZ2P SR	1132.66	−1272.50	342.64	−1614.89	NA
PBE0 TZ2P SOR	1168.15	−1322.45	341.44	−1641.21	−22.68
PBE0 HF40 TZ2P SR	1371.56	−1539.91	343.29	−1883.20	NA
PBE0 HF40 TZ2P SOR	1357.21	−1525.88	343.47	−1859.81	−9.54
SAOP SR	612.10	−763.60	342.54	−1106.13	NA
SAOP SOR	621.65	−772.93	342.50	−1084.46	−31.00
B3LYP TZ2P SO	1040.01	−1228.58	344.59	−1573.17	NA
B3LYP TZ2P SOR	1044.23	−1232.8	344.73	−1546.11	−31.43

Supplementary Table 3. Experimental and calculated solution $^{15}N_{nitride}$ δ_{iso} values to derive the Empirical Scaling factor using the B3LYP functional. Value for **1*** is in THF.

**Compound**	**δ_{iso}(exp)**	**δ_{iso}(calc)**
**1***	969	1044
**MoN**	460	539
**$MoNBF_3$**	212	240
**TiN**	578	613
**TiNK**	542	611

Supplementary Table 4. Experimental and calculated solid-state $^{15}N_{nitride}$ δ_{iso} values to derive the Empirical Scaling factor using the B3LYP functional.

**Compound**	**δ_{iso}(exp)**	**δ_{iso}(calc)**
**1***	950	1105
**MoN**	453	539
**$MoNBF_3$**	213	240
**TiN**	522	613
**TiNH**	536	−13

Supplementary Table 5. Computed HOMO-LUMO gap, mean, median, and range values for the complexes computed in this study with BP86, PBE0, and B3LYP functionals. Values are all in eV.

Compound	BP86	PBE0	B3LYP
**1***	1.80	4.21	3.70
**VN(O)**	2.28	4.05	3.80
**VN(N)**	1.78	3.71	3.34
**TiN**	1.92	3.97	3.61
**TiNK**	1.83	3.91	3.59
**MoN**	2.82	4.82	4.39
**$MoNSnCl_2$**	2.57	4.24	3.99
**$MoNGeCl_2$**	2.51	4.04	3.92
**$MoNBF_3$**	2.63	4.54	4.08
**$MoNSiMe_3$**	2.32	4.10	3.66
**MoNCOPh**	1.97	3.54	3.08
**MoNEt**	2.42	4.22	3.81
**$MoNCH_2$**	1.40	3.24	2.86
**TiNH**	1.84	3.46	3.19
**ZrNH**	1.75	3.74	3.35
**ThNTh**	3.48	5.42	5.18
**$ThNH_2$**	3.74	6.01	5.62
**Mean**	2.30	4.19	3.83
**Median**	1.97	4.04	3.66
**Range**	1.99	2.77	2.76

Supplementary Table 6. Experimental solution $^{15}N_{nitride}$ δ_{iso} and computed Mayer, NM, DI, and V_{XC} data.

Compound[a]	δ_{iso}	Mayer	NM	DI	V_{XC}
**1*$_{nitride}$**	[d] 971	2.94	3.54	2.649	–0.607
**1*$_{amide}$[b]**	–123	0.91	1.33	0.790	–0.158
**1*$_{amine}$[b]**	–311	0.44	0.5	0.377	–0.077
**MoN**	460	2.64	2.72	2.512	–0.558
**$MoNBF_3$**	212	1.95	2.22	2.188	–0.489
**$MoNGeCl_2$[c]**	263	2	2.26	2.175	–0.485
**$MoNSnCl_2$[c]**	286	2.14	2.34	1.531	–0.391
**$MoNSiMe_3$**	157	1.69	2.08	1.944	–0.430
**MoNCOPh[c]**	87	1.43	1.81	1.737	–0.389
**MoNEt[c]**	80	1.72	1.97	1.886	–0.423
**$MoNCH_2$**	75	1.41	1.74	1.606	–0.365
**TiNK**	542	2.55	2.88	2.116	–0.446
**TiN**	578	2.69	2.98	2.216	–0.469
**TiNH**	41	1.89	2.4	1.694	–0.359
**ZrNH**	–29	1.58	2.3	1.466	–0.306
**ThNTh**	299	1.31	1.68	1.143	–0.238
**$ThNH_2$**	–198	0.6	1.27	0.763	–0.158
**VN(O)**	679	2.85	3.06	2.583	–0.570
**VN(N)**	655	2.86	3.02	2.555	–0.564

[a] All values referenced to $MeNO_2$ = 0 ppm. [b] Computed but correlated values. [c] Where solution data are absent we have used solid-state δ_{iso} values noting that for these Mo complexes where solution and solid-state ^{15}N data are available the difference between phases is <7 ppm. [d] Average value.
